# A SNARE-Like Superfamily Protein *SbSLSP* from the Halophyte *Salicornia brachiata* Confers Salt and Drought Tolerance by Maintaining Membrane Stability, K^+^/Na^+^ Ratio, and Antioxidant Machinery

**DOI:** 10.3389/fpls.2016.00737

**Published:** 2016-06-02

**Authors:** Dinkar Singh, Narendra Singh Yadav, Vivekanand Tiwari, Pradeep K. Agarwal, Bhavanath Jha

**Affiliations:** ^1^Division of Marine Biotechnology and Ecology, CSIR-Central Salt and Marine Chemicals Research InstituteBhavnagar, India; ^2^Academy of Scientific and Innovative ResearchCSIR, New Delhi, India

**Keywords:** abiotic stress, SNARE-like superfamily protein, clathrin adaptor protein complex, clathrin-coated vesicles, halophyte, *Salicornia brachiata*, salt-inducible gene

## Abstract

About 1000 salt-responsive ESTs were identified from an extreme halophyte *Salicornia brachiata*. Among these, a novel salt-inducible gene *SbSLSP* (S*alicornia* b*rachiata* SNARE-like superfamily protein), showed up-regulation upon salinity and dehydration stress. The presence of *cis*-regulatory motifs related to abiotic stress in the putative promoter region supports our finding that *SbSLSP* gene is inducible by abiotic stress. The SbSLSP protein showed a high sequence identity to hypothetical/uncharacterized proteins from *Beta vulgaris, Spinacia oleracea, Eucalyptus grandis*, and *Prunus persica* and with SNARE-like superfamily proteins from *Zostera marina and Arabidopsis thaliana*. Bioinformatics analysis predicted a clathrin adaptor complex small-chain domain and N-myristoylation site in the SbSLSP protein. Subcellular localization studies indicated that the SbSLSP protein is mainly localized in the plasma membrane. Using transgenic tobacco lines, we establish that overexpression of *SbSLSP* resulted in elevated tolerance to salt and drought stress. The improved tolerance was confirmed by alterations in a range of physiological parameters, including high germination and survival rate, higher leaf chlorophyll contents, and reduced accumulation of Na^+^ ion and reactive oxygen species (ROS). Furthermore, overexpressing lines also showed lower water loss, higher cell membrane stability, and increased accumulation of proline and ROS-scavenging enzymes. Overexpression of *SbSLSP* also enhanced the transcript levels of ROS-scavenging and signaling enzyme genes. This study is the first investigation of the function of the *SbSLSP* gene as a novel determinant of salinity/drought tolerance. The results suggest that *SbSLSP* could be a potential candidate to increase salinity and drought tolerance in crop plants for sustainable agriculture in semi-arid saline soil.

## Introduction

The human population is growing rapidly and expected to be more than 9 billion by 2050 (Godfray et al., 2010). In the mission to meet food demand for the ever-increasing world population, adverse environmental factors are becoming a major challenge for the scientific community. The environmental factors affecting crop yield are mainly grouped as biotic and abiotic stresses. Salinity, drought, high temperature, waterlogging, high light intensity and the mineral deficiency are the major abiotic stresses which negatively affects plant growth, resulting in reduced yield or may leads to death of the plants at extreme conditions (Mahajan and Tuteja, 2005). These stresses are responsible for reducing yield by more than 50% of major crops across the globe (George et al., 2012). Plants respond to environmental cues via activation of various defense mechanisms that include environmental stress signal perception, transduction pathways and, upregulation/downregulation of numerous genes, and synthesis of protective molecules (Zhu, 2002; Huang et al., 2011). The roles of many stress responsive genes and micro RNAs and few of the *cis*- and *trans*- regulatory factors have been studied in stress tolerance (Yamaguchi-Shinozaki and Shinozaki, 2006; Agarwal and Jha, 2010; Nakashima et al., 2012; Singh and Jha, 2014).

Most of the earlier studies to understand the salinity tolerance mechanism have been focused on glycophytes, except some of the recent studies that include halophytes (Agarwal et al., 2013). The halophytes have unique genetic makeup (Agarwal et al., 2010), which accounts for its better tolerance mechanisms to thrive in saline environment than the glycophytes (Gong et al., 2005) and thus emerged as model organism to study the salinity tolerance mechanism in plants.

The expressed sequence tag (EST) database served as potential resource to identify novel gene(s) contributing to specific biological processes (Jha et al., 2009; Yadav et al., 2012a). The EST database of different plant species showed a major portion of unknown/ hypothetical genes that lack any similarity with known genes in the NCBI database (Yadav et al., 2014). The unknown/hypothetical novel genes identified from the EST databases can be chosen for crop genetic engineering for enhanced stress tolerance. Recently, several genes from different halophytes have been reported to increase the salinity as well as drought stress tolerance in crop, tobacco or Arabidopsis (Himabindu et al., 2016 and references therein). For example, Li et al. (2011) has found that the transgenic alfalfa plants overexpressing *Salsola soda NHX1* (*SsNHX1)* has better salinity tolerance and survive well for 50 days in the soil irrigated with 400 mM saline solution twice a week. Transgenic hybrid poplar plants overexpressing *Populus trichocarpa PtSOS2* showed increased plasma membrane Na^+^ exclusion activity and better ROS scavenging compared to WT plants (Yang et al., 2015). Not only the transporter genes, overexpression of a halophytic *ASR1* gene and NAC transcription factor into *Arabidopsis thaliana* which are cloned from *Suaeda liaotungensis* had improved the salinity, drought, and low-temperature tolerance (Hu et al., 2014; Li et al., 2014).

*Salicornia brachiata* Roxb., an extreme annual halophyte grows in saline (0.1–2.0 M) coastal land and can store salt as high as 40% of its dry weight (Tiwari et al., 2015). These unique characteristics of this plant may enable it as a potential model plant to study salt-responsive genes. Earlier, we characterized some of the genes including *SbMAPKK* (Agarwal et al., 2010), *SbDREB2A* (Gupta et al., 2010), *SbGST* (Jha B. et al., 2011), *SbNHX1* (Jha A. et al., 2011), *SbASR1* (Jha et al., 2012), *SbMT-2* (Chaturvedi et al., 2012), *SbSI-1* (Yadav et al., 2012a), *SbSOS1* (Yadav et al., 2012b), *SbUSP* (Udawat et al., 2013, 2016), and *SbSI-2* (Yadav et al., 2014) from the EST database of *S. brachiata* (Jha et al., 2009) and confirmed their role in abiotic stress tolerance. Therefore, these genes may serve as potential candidate for bioengineering of other crops to enhance their abiotic stress tolerance. In light of the above facts, here we have cloned and characterized another salt-inducible gene *SbSLSP* and its promoter from *S. brachiata*. Bioinformatics analysis predicted abiotic stress related *cis*-regulatory motifs in promoter region, which is further confirmed with higher transcript expression of *SbSLSP* in salt and drought stress. Subcellular localization studies indicated that the SbSLSP protein is mainly localized in the plasma membrane. Further we confirmed *SbSLSP* role in stress tolerance through a transgenic approach in tobacco plants. The enhanced tolerance of transgenic tobacco was established by various morphological, physiological and biochemical parameters. Overexpression of *SbSLSP* also enhanced the transcript levels of ROS-scavenging and signaling enzyme genes. This study provides novel insights into the function of *SbSLSP* gene in response to salinity and drought stress.

## Materials and methods

### Plant growth and stress treatments

The *Salicornia brachiata* Roxb. seeds were harvested from dried plants collected from the coastal area near Bhavnagar (latitude 21° 45′ N, longitude 72° 14′ E), Gujarat, India. The seeds were germinated in plastic pots containing garden soil, and the plants were grown in natural conditions. One-month-old seedlings were carefully uprooted and transferred to hydroponic culture ½ strength Murashige and Skoog (MS) salts (Murashige and Skoog, 1962) in a culture room with a dark/light cycle of 8/16 h at 25°C for 1 month. The nutrient solution was renewed twice a week. Plants were given stress treatment using 250 mM NaCl in ½ MS solution, and desiccated by wrapping the root in tissue paper for 0, 6, 12, and 24 h. Upon completion of the treatments, shoot tissues were collected, frozen in liquid nitrogen and stored at −80°C.

### Cloning of the *SbSLSP* gene and its putative promoter

The EST of *SbSLSP* was made full length and characterized for its role in abiotic stress tolerance. The total RNA was extracted from shoot tissues of the salt-stressed plants of *S. brachiata* by the GITC method (Chomczynski and Sacchi, 1987). The 5′-RACE reaction and PCR amplification was performed using primers GSPR1, GSPR2, GSPR3, AAP, and AUAP according to the manufacturer's protocol (Invitrogen, USA). The amplicon was purified from agarose gel and cloned into the pGEM-T Easy vector system II (Promega, Madison, Wisconsin) and sequenced (Macrogen Inc., Seoul, South Korea). After determining the open reading frame (ORF), the full-length *SbSLSP* cDNA was PCR-amplified with AccuPrime Pfx DNA polymerase (Invitrogen, USA) in conjunction with primers SbSLSPF and SbSLSPR containing *Hind*III and *Xho*I sites, respectively. The amplification product was then cloned into a pJET1.2/blunt cloning vector (MBI Fermentas) and sequenced. The gene sequence was submitted to the NCBI database (accession number KF111691).

Three gene-specific primers named SbSLSPPR1, SbSLSPPR2, and SbSLSPPR3 were designed from the cDNA sequence. Then, the putative promoter region was isolated using the genome-walking technique (Tiwari et al., 2014, 2016). The putative promoter was sequenced and submitted to NCBI (accession number KP229524). See Table S1 for primer sequences.

### *In silico* analysis

The NCBI database was used as a search engine for nucleotide and protein sequences. TMpred online software was used for the prediction of transmembrane domains and ClustalW software was used for sequence alignment. Conserved domains of SbSLSP were determined by BLASTp programme (http://www.ncbi.nlm.nih.gov). Secondary structure prediction of SbSLSP was carried out by Expasy tools (http://www.expasy.ch/tools/), while the phosphorylation motifs were predicted by NetPhosK 1.0 server. The Pfam and SMART programs were used for conserved domain analysis. The relationship of SbSLSP with its homologs from other plant species was inferred by constructing a phylogenetic tree using MEGA Ver. 6.1. The robustness in topology of the phylogenetic tree was assessed based on bootstrap values. *In silico* analysis of the putative promoter fragment was performed for the presence of *cis*-regulatory elements using the online programs PLACE (http://www.dna.affrc.go.jp/PLACE) and PlantCARE (http://bioinformatics.psb.ugent.be/webtools/plantcare/html/).

### Quantitative real-time PCR (qRT-PCR) analysis

Total RNA was isolated from *S. brachiata* control and treated plant samples using the GITC method (Chomczynski and Sacchi, 1987) and qRT-PCR of *SbSLSP* was performed as previously described Yadav et al. (2014) using primer pair SbSLSP_RTF1 and SbSLSP_RTR1. The β*-tubulin* gene was used as an internal control and ampliflied using primers BTF and BTR. Samples were amplified in triplicate and independent experiments were repeated three times. Fold changes were calculated using the 2${}^{-\Delta \Delta {\text{C}}_{\text{T}}}$ method (Livak and Schmittgen, 2001). See Table S1 for primer sequences.

### Subcellular localization of SbSLSP protein

The localization fusion construct with RFP (red fluorescent protein) was made using Gateway technology. The full-length *SbSLSP* cDNA was PCR-amplified with AccuPrime Pfx DNA polymerase in conjunction with SbSLSPCAF and SbSLSPCAR primers. The blunt-ended PCR product was then cloned into a *pENTER/D-TOPO* entry vector and then into the destination vector *pSITE-4CA* by Gateway LR Clonase II enzyme mix (Yadav et al., 2014; Invitrogen, USA). The fusion construct (*RFP:SbSLSP*) was transferred into onion epidermal cells by particle bombardment (PDS-1000/He Biolistic, Biorad, USA). The *pSITE-4CA* (RFP) vector was used as a control. After incubation on an MS plate for 12–24 h, the onion epidermal cells were observed for transient expression of RFP with an epifluorescence microscope (Axio Imager, Carl Zeiss AG, Germany). See Table S1 for primer sequences.

### Construction of plant transformation vector and tobacco transformation

To perform plant transformation, *SbSLSP* cDNA was PCR-amplified with AccuPrime Pfx DNA polymerase in conjunction with SbSLSPPF and SbSLSPPR primers that contained *ApaI* and *KpnI* sites, respectively. The *SbSLSP* gene was cloned into the pRT100 vector (Topfer et al., 1987) to attach the *35S* promoter and terminator. The above gene expression cassette (*35S-SbSLSP*-terminator) was sub-cloned into the *pCAMBIA2301* binary vector at the *PstI* site. The resultant construct *pCAMBIA2301-35S:SbSLSP* and *pCAMBIA2301* alone were mobilized into *Agrobacterium tumefaciens* (LBA 4404) for tobacco (*Nicotiana tabacum* var. Xanthi) transformation (Horsch et al., 1985). Independently regenerated and kanamycin resistant (50 mg/l) transgenic lines (T_0_) were propagated and screened using GUS assay (β-Glucuronidase Reporter Gene Staining Kit; Sigma-Aldrich) and PCR analysis (Yadav et al., 2014). By selection with resistance to kanamycin (100 mg/l) at the seedling stage of T_1_ and T_2_ plants, were harvested for subsequent experiments. At T_1_ generation, lines which showed 3:1 ratio of resistant to sensitive on the MS plates supplemented with kanamycin (100 mg/L) were selected and grown to harvest the seed of next generation. At T_2_ generation, the lines which seeds shown 100% resistance on antibiotic plates was chosen for the further analysis. See Table S1 for primer sequences.

### Molecular analysis of transgenic lines

The presence of the transgene was confirmed by PCR using a *SbSLSP*-specific primer pairs SbSLSP_RTF2 and SbSLSP_RTR2. To confirm the expression of GUS activity, histochemical staining was conducted as described previously (Yadav et al., 2014). To check the transcript expression of the overexpressed *SbSLSP* gene in transgenic plants, reverse-transcriptase PCR (RT-PCR) was carried out. Total RNA was isolated from wild-type (WT) and transgenic plant samples, and cDNA was prepared. The RT-PCR was performed as previously described (Yadav et al., 2014) using the *SbSLSP*-specific primer pair. The primer pair QACTF and QACTR was used for *actin* as the internal control. See Table S1 for primer sequences.

### Evaluation of transgenic plants exposed to salt and osmotic stress

To analyse the stress tolerance of *SbSLSP*-overexpressing tobacco plants (T_2_), the seeds were germinated on MS agar medium supplemented with 200 mM NaCl (salt stress) or 300 mM mannitol (osmotic stress) in culture room conditions. The percentage of seed germination was scored for 9 days at 2-day intervals. For study of seedling root morphology, the germinated seeds of transgenic and WT plants were grown on vertical plates for 10 days in MS media and 21 days for MS media supplemented with 200 mM NaCl or 300 mM mannitol. The primary root lengths and fresh weights were recorded, and physiological changes were observed at the end of the culture periods. For salinity and drought tolerance assays in plant, T_2_ transgenic lines and WT seeds were germinated on MS medium. Ten-day-old seedlings were planted in identical pots containing soilrite mix (horticulture-grade expanded perlite, Irish Peat moss and exfoliated vermiculite in equal ratio) and were well watered with 1/8 MS salt solution for 20 more days in the growth chamber (22°C, 60% humidity, 16 h light/8 h dark cycle). The transgenic and WT plants with similar size and growth were divided into four groups. Plants were watered every 2 days with 1/8 MS salt solution supplemented with (three groups) or without (one group) 250 mM NaCl for 18 days. In another set of drought stress assays, plants irrigation was stopped for 18 days and recovered after re-watering for next 5 days. During these treatments, morphological changes were observed and photographed, and the survival rate was measured. The leaves, stems and roots of each plant were harvested separately for Na^+^ and K^+^ determination. Leaf-disc assays to determine the total chlorophyll content of transgenic and WT plants were performed with protocols as described in earlier reports (Porra et al., 1989; Chaturvedi et al., 2014). In another experiment, 6-week-old plants were subjected to ¼ MS salt solution containing NaCl (250 mM) or PEG-6000 (20%, w/v) stress in hydroponic conditions for 3 weeks. MS salt solution containing NaCl and PEG were replaced every second day during the stress assay, and photographs were taken. Consistent results were observed in three independent experiments. The histochemical detection of H_2_O_2_ in the leaves of NaCl- or PEG-treated plants for 12 h was measured as described by Mukherjee and Choudhuri (1983). All experiments were repeated with three experimental and biological replicates. Each biological replicate contained 10 plants.

### Determination of Na^+^ and K^+^ content

Plant materials collected at the end of salt treatment were used for determination of Na^+^ and K^+^ content via the method previously described by Shukla et al. (2012).

### Measurement of various biochemical and physiological parameters

Three-week-old seedlings were subjected to salinity (250 mM NaCl) or osmotic (20% (w/v) PEG-6000) stress for 1, 6, 12, and 24 h. Cell membrane stability (CMS), relative water content (RWC), proline, and H_2_O_2_ content were measured from fresh samples after stress recovery (Bates et al., 1973; Wang et al., 2011; Garg et al., 2012). MDA and activity of antioxidant enzymes (APX: EC 1.11.1.11 and GR: EC 1.6.4.2) were performed with stressed samples stored at −80°C (Garg et al., 2012). Ten seedlings were pooled as one sample, and three replicates of samples were taken for all lines in each experiment.

### Expression analysis of reactive oxygen species (ROS)-scavenging and signaling genes

The expression patterns of ROS-scavenging (*NtSOD:* EC 1.15.1.1, *NtAPX:* EC 1.11.1.11, *NtPOX:* EC 1.11.1.7) and signaling (*NtPLC1*) genes in transgenic and WT plants were also analyzed by qRT-PCR as described previously (Yadav et al., 2014). The gene-specific primer pairs of *NtSOD, NtAPX, NtPOX* (primer sequences taken from Huang et al., 2011) and phosphoinositide-specific phospholipase C1 *NtPLC1* (NtPLCF and NtPLCR primers) were utilized for the expression study. The *actin* gene was used as an internal control and amplified using primers QACTF and QACTR to normalize all data. Samples were amplified in triplicate, and independent experiments were repeated three times. Fold changes were calculated using the 2${}^{-\Delta \Delta {\text{C}}_{\text{T}}}$ method (Livak and Schmittgen, 2001). See Table S1 for primer sequences.

### Statistical analyses

One-way ANOVA using between subject factors algorithm was performed by ezANOVA (http://www.cabiatl.com/mricro/ezanova/) programme for analysis of variance to determine the least-significant difference among means. Mean values that were significantly different within treatment group were evaluated by Duncan's test at ^*^P < 0.05, ^**^P < 0.01 and ^***^P < 0.001.

## Results

### Isolation and sequence analysis of *SbSLSP* and its promoter

The EST of *SbSLSP* (GenBank accession number EB484673) was made full length using 5′-RACE. The cDNA of *SbSLSP* (NCBI accession id KF111691) was 840-bp long, including a 5′ UTR of 60-bp, an ORF of 444-bp and a 3′ UTR region of 336-bp (Figure S1). The encoded protein of *SbSLSP* contains 147 amino acids with an estimated molecular weight of 16.84 kDa and a pI of 5.44. The NCBI protein blast (blastp) analysis revealed that the SbSLSP protein has a high sequence identity (78–95%) to a SNARE-like superfamily protein and/or hypothetical uncharacterized proteins from different plant species. It has 78 and 84% sequence identity with *A. thaliana* and *Zostera marina* SNARE-like proteins, respectively. The SbSLSP also showed high identity with uncharacterized/ hypothetical proteins of *Beta vulgaris* (95%), *Spinacia oleracea* (93%), *Eucalyptus grandis* (85%), and *Prunus persica* (84%). Phylogenetic analysis revealed that SbSLSP showed close neighborhood with other Amaranthaceae family members *B. vulgaris* and *S. oleracea*, which is suggesting the evolutionary conservation within the family. The SbSLSP also showed close proximity with other halophyte *Z. marina* (Figure S2). The bioinformatics analysis using the Pfam program predicted a clathrin adaptor complex small-chain domain in the SbSLSP protein. The SbSLSP protein may be phosphorylated by CKII (caseine kinase II) and PKC (protein kinase C). The SbSLSP also contained an N-myristoylation site at the 125–130 amino acid position (GVleNT) (Figure S1). There was no transmembrane domain predicted in SbSLSP by hydropathicity analysis, whereas the SbSLSP secondary structure analysis predicted four, six and eleven alpha helixes, extended strands and random coils, respectively (Figure S3). The 872-bp putative promoter region upstream of translation start site ATG had a number of *cis*-regulatory motifs that were categorized into six different groups: abiotic stress inducible, conserved motifs, light responsive, miscellaneous, phytohormone inducible and tissue/organelle-specific expression (Table S2). Sequence analysis revealed the presence of several *cis*-regulatory motifs related to abiotic stress responses, like anaerobic, drought, metal, salinity, and high temperature stresses, in the promoter region (Table S3; Figure S4).

### Differential expression of *SbSLSP* transcripts under salinity and desiccation stress

**Figure 1:**
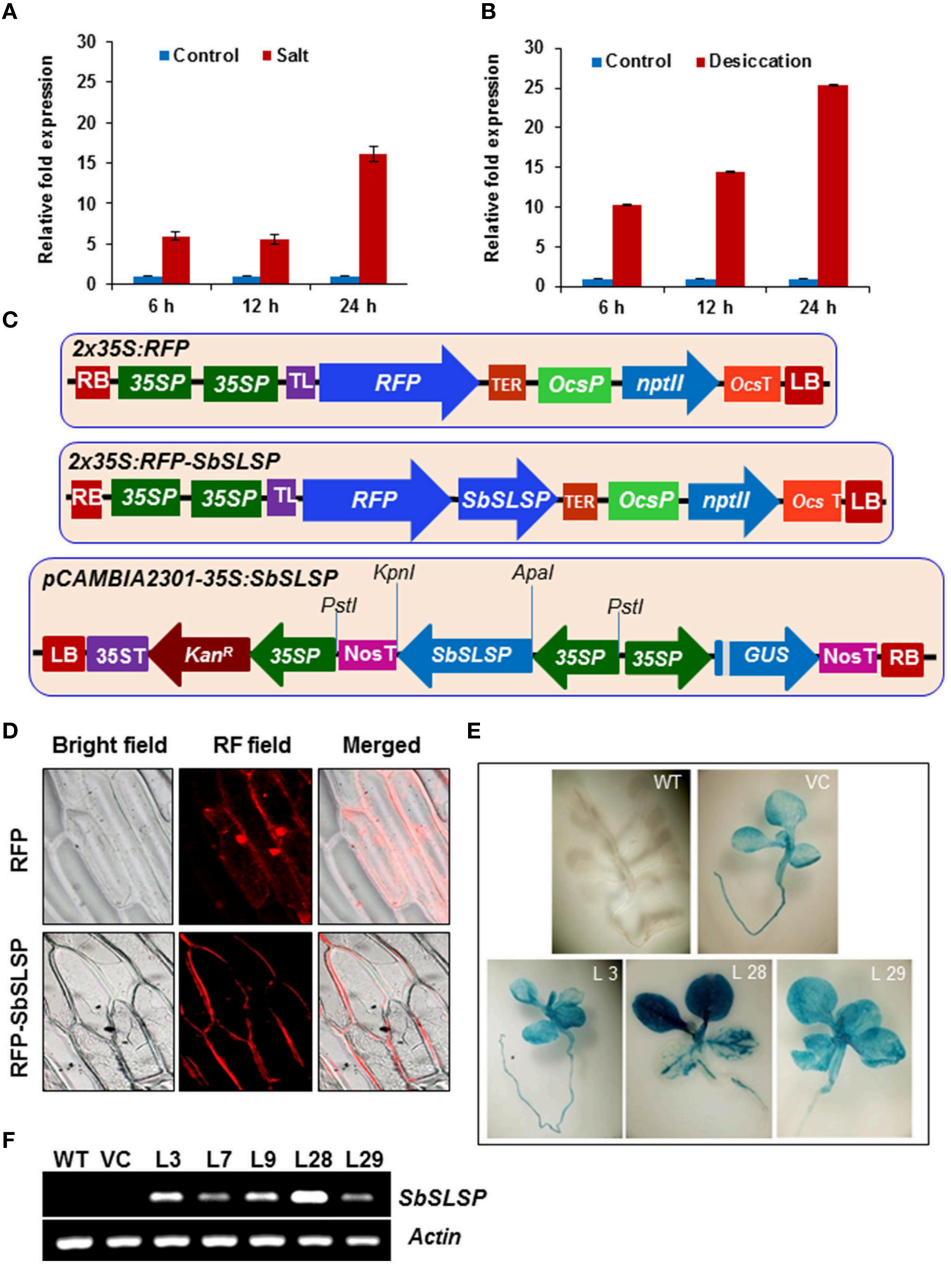
**Expression, localization and molecular analyses of ***SbSLSP***–overexpressing transgenic tobacco plants**. **(A,B)** Expression pattern of *SbSLSP* in response to salt and desiccation stress treatments for different time periods in *Salicornia brachiata*. The relative fold expression of *SbSLSP* in stressed and non-stressed conditions was calculated using the 2${}^{-\Delta \Delta {\text{c}}_{\text{T}}}$ method. Values are means ± SD from three independent measurements. **(C)** Schematic representation of the *pSITE-3CA-2X35S:RFP:SbSLSP* construct (*RFP:SbSLSP*) used for transient expression and *pCAMBIA2301-35S:SbSLSP* construct used to transform tobacco plants with the *SbSLSP* gene. **(D)** SbSLSP resides in the plasma membranes of onion epidermal cells. Cells with constructs expressing red fluorescence protein (RFP) alone and the RFP:SbSLSP fusion protein were analyzed using a bright and red fluorescence field microscope. **(E)** GUS assay of T_2_ seedlings showing positive GUS expression in the transgenic lines. **(F)** Transcript levels of the *SbSLSP* gene in transgenic lines and WT T_2_ plants via semi-quantitative RT-PCR.

The expression profile of the *SbSLSP* gene was carried out by qRT-PCR in *S. brachiata*. In the presence of 250 mM NaCl, the transcript of *SbSLSP* increased by 6-fold at 6 h and it remain unchanged at 12 h and then increased up to 16-fold at 24 h time-point of stress treatment (Figure 1A). In desiccation stress, the transcript was up-regulated from 10- to 25-fold and the maximum expression was observed at 24 h time-point (Figure 1B).

### The SbSLSP protein is localized to the plasma membrane

The transient expression assay into onion epidermal cells was performed to determine the *in vivo* subcellular localization of SbSLSP using RFP alone or the *RFP:SbSLSP* fusion construct into *pSITE-4CA* vector (Figure 1C). Onion cells were transformed with *RFP* alone, displayed even distribution of red fluorescence signals in the entire cell region, whereas in *RFP:SbSLSP* transformed cells, the fluorescence was predominantly accumulated in the plasma membrane and only slightly in the cytoplasmic region (Figure 1D). These results clearly indicate that SbSLSP protein is mostly localized in the plasma membrane and to some extent in the cytoplasmic region.

### Analysis of *SbSLSP*–overexpressing transgenic tobacco plants

The tobacco plants were genetically transformed with *pCAMBIA2301-35S:SbSLSP* construct (Figure 1C) and *pCAMBIA2301* empty vector for *in vivo* functional characterization of the *SbSLSP*. Total 35 independent transgenic lines which were resistant to kanamycin were also confirmed by PCR using gene-specific as well as *gus*-specific primers (data not shown). The empty vector-transformed transgenic (VC) and WT plants were used as controls. The germination assay of seeds from these T_1_ transgenic plants on kanamycin-supplemented medium exhibited the ratio of 3:1 (Kan^r^/Kan^s^). The T_2_ generation lines were again verified by PCR amplification. At T_2_ generation, the three *35S:SbSLSP* GUS-positive and one empty-vector transgenic lines (L3, L28, L29, VC) were chosen for subsequent analysis (Figure 1E). The *SbSLSP*–overexpressing transgenic lines exhibited different levels of *SbSLSP* expression via RT-PCR, whereas there were no expression of *SbSLSP* was detected in WT and VC plants (Figure 1F). The transgene transcript expression detected in L28 transgenic line was maximum among other lines used in the analysis (Figure 1F).

### Salinity and osmotic stress responses of *SbSLSP*–overexpressing transgenic lines

**Figure 2:**
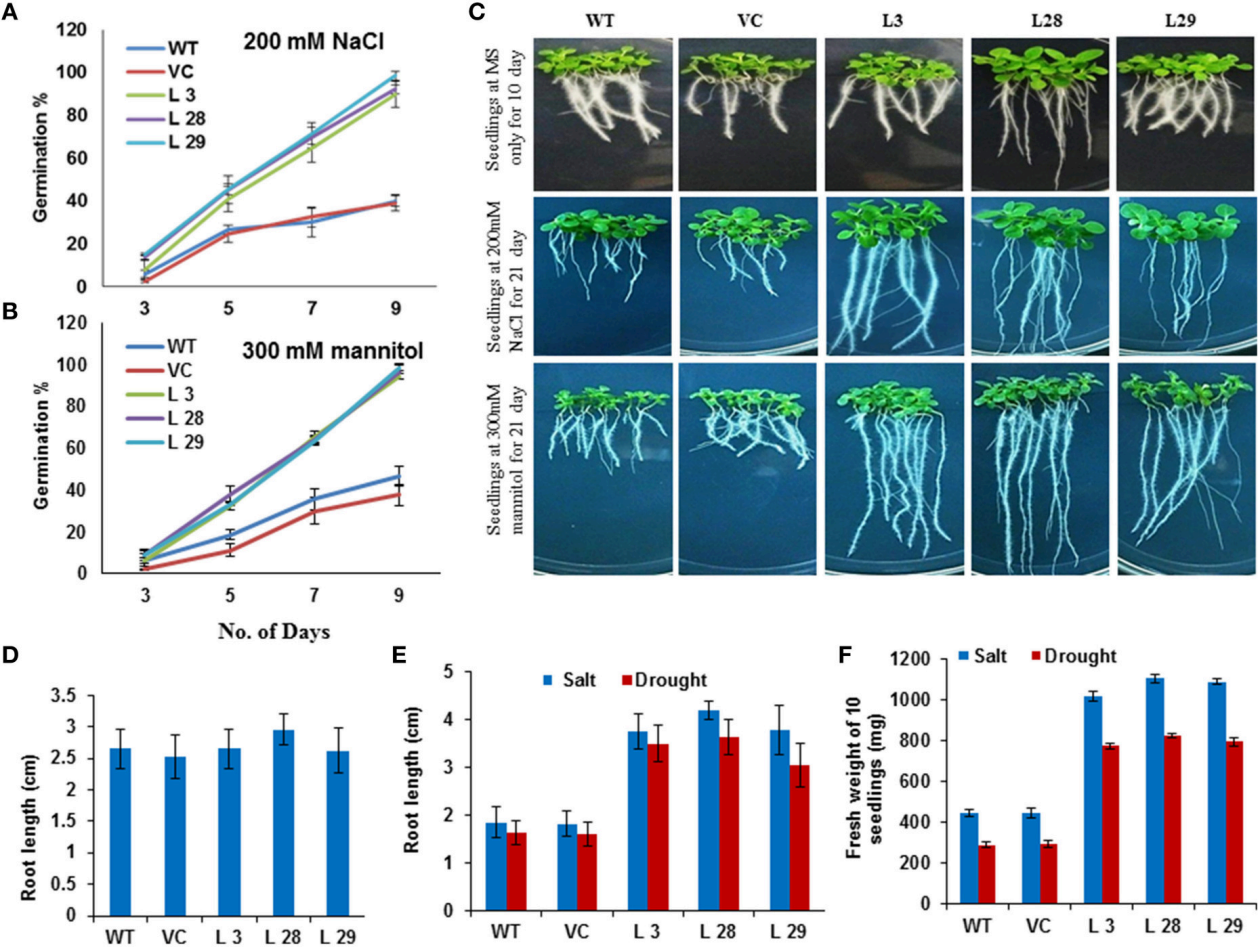
**Effect of ***SbSLSP*** on stress tolerance in T_**2**_ transgenic tobacco**. **(A,B)** Germination of *SbSLSP* transgenic and wild-type plants in MS medium supplemented with 200 mM NaCl or 300 mM mannitol. The percentage of germinated seeds was calculated based on the number of seedlings that reached the cotyledon stage by 9 days. **(C)** Root growth comparisons of the germinated seedlings of transgenic and WT plants, which were grown on vertical plates for 10 days in MS media for control experiment and 21 days for MS media supplemented with 200 mM NaCl or 300 mM mannitol for stress experiment. **(D,E)** Graphical representation of root measurements of transgenic and wild-type plants in control and stress conditions (salt or drought stress), respectively. Seeds of each line were germinated and planted on MS agar, or MS agar supplemented with 200 mM NaCl or 300 mM mannitol in triplicate. Root length was measured at 10 days for MS agar and 21 days for MS agar supplemented with 200 mM NaCl or 300 mM mannitol. Values are the mean ± SD (triplicate measurements; *n* = 10). **(F)** Fresh weight measurement of 21-day-old seedlings in salt and drought stress. Values are the mean ± SD (triplicate measurements; *n* = 10).

The effect of salinity and osmotic stress on germination was studied by germinating transgenic lines and WT seeds on MS medium comprising 200 mM NaCl or 300 mM mannitol. In the medium containing NaCl, the maximum germination rate for WT and VC were 40 and 39%, respectively, while for overexpressing lines, more than 89% germination was recorded (Figure 2A). In the medium containing mannitol, the maximum germination rate for WT and VC was 46.8 and 37.5%, respectively, while the germination rate was more than 92% for transgenic lines (Figure 2B). In addition to seed germination assays, physiological changes were observed. The seedlings of WT, VC and overexpressing lines were shifted to MS medium with or without 200 mM salt or 300 mM mannitol. We measured the primary root length after 10 days, and observed that primary roots were almost equal in all the lines growing at ½ MS (Figures 2C,D). The physiological changes of the T_2_ transgenic seedlings, compared to the WT seedlings, were observed after 21 days of salinity and osmotic stresses, and *SbSLSP*–overexpressing lines showed significantly better growth performance (Figure 2C). After 21 days, most of the WT and VC plant growth had stopped, while the *SbSLSP*–overexpressing lines showed significantly higher root and shoot growth (Figures 2C,E). Furthermore, the fresh weight analysis showed that salinity and osmotic stress had low adverse effect on the *SbSLSP*–overexpressing lines, while significant growth impairment was seen in the biomass synthesis of WT and VC plants (Figure 2F).

### Analysis of *SbSLSP*–overexpressing plants (T_2_) in salt and drought stress

**Figure 3:**
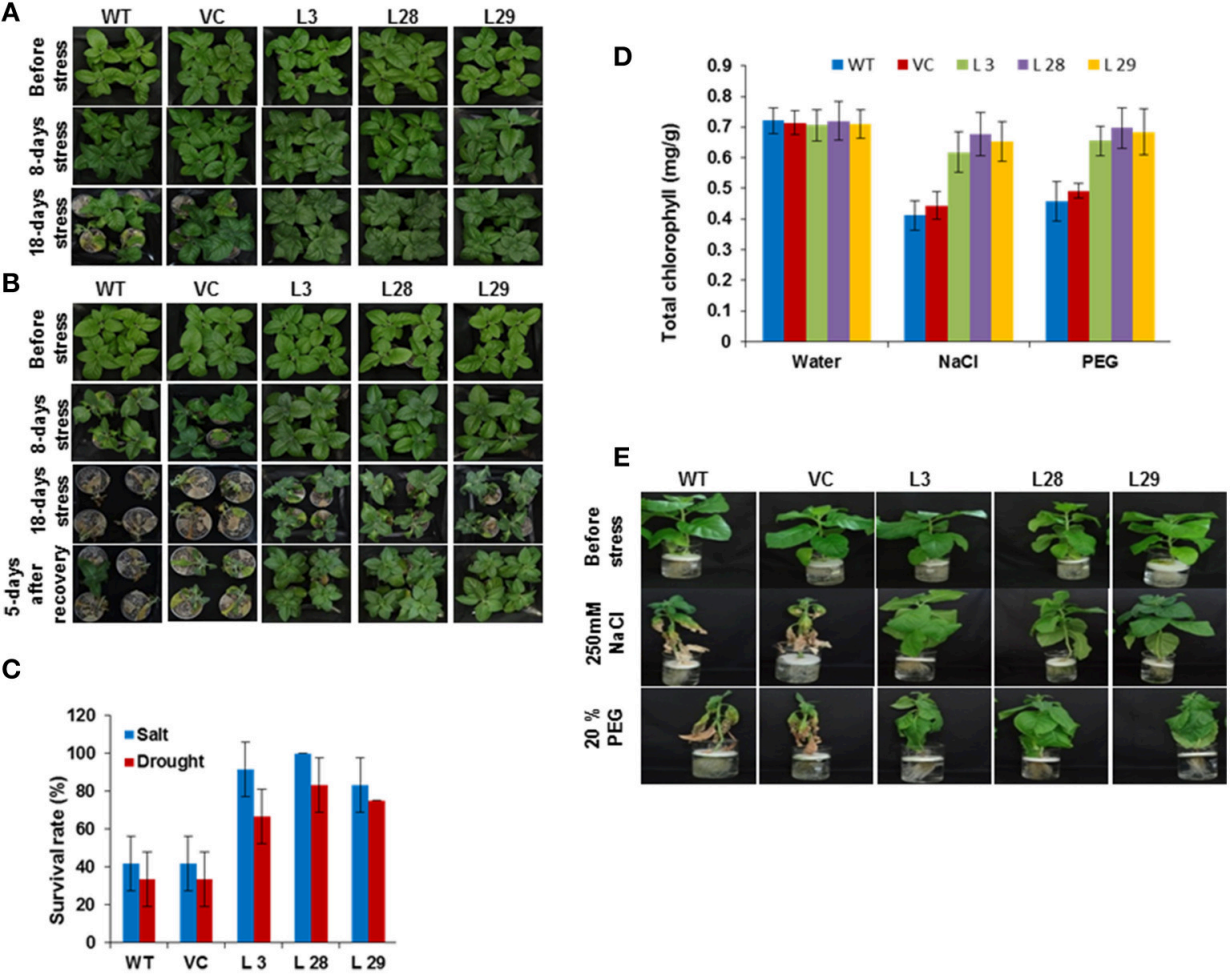
**Response of wild-type (WT), vector control (VC), and ***SbSLSP*** T2 transgenic tobacco plants under salt and drought stress**. **(A)** Five-week-old transgenic lines (L 3, L 28, L 29, VC) and WT were irrigated with NaCl solution (250 mM) for 18 days. Photographs of representative seedlings were taken at the initial stage of salt stress, 8 days after stress, and 18 days after stress. **(B)** The water supply of 5-week-old normal-growing transgenic lines (L 3, L-28, L-29, VC) and WT was depleted 18 days. Photographs of representative seedlings were taken at the initial stage of drought stress, 8 days after stress, 18 days after stress, and 5 days after re-watering. **(C)** Quantitative analysis of survival of transgenic lines and WT plants during stress. Values are the mean ± SD (triplicate measurements; *n* = 4 plants). **(D)** Quantification of chlorophyll retention from the leaf-disc assay. Values are the mean ± SD of three independent assays. **(E)** Representative photographs of 6-week-old transgenic plants (L 3, L 28, L 29, VC) grown in 250 mM NaCl or 20% PEG compared to WT for 3 weeks.

To characterize the performance of *SbSLSP*–overexpressing plants under salinity and drought stress, 10-day-old seedlings were grown in Soilrite for an additional 20 days. Thereafter, plants were treated with 250 mM NaCl for 18 days to induce salt stress and irrigation was stopped for 18 days to induce drought stress. The signs of stress, such as wilting symptoms, were more evident in WT and VC than those of the *SbSLSP*–overexpressing plants for both the stresses (Figures 3A,B). In the presence of salinity stress, more than 83% of *SbSLSP*–overexpressing plants survived, compared to only 41% survival for WT and VC plants (Figure 3C). Five days after re-watering to drought stressed plants, 65–83% of *SbSLSP–*overexpressing plants survived, whereas the survival rate was 33% for WT and VC plants (Figure 3C). We also performed leaf-disc assays to determine chlorophyll content under stress-induced necrosis of leaf disc. The transgenic plants retained higher chlorophyll contents as compared to WT and VC plants under stress treatments (Figure 3D). To evaluate the stress effects on the growth of *SbSLSP*–overexpressing plants, 6-week-old plants were grown in hydroponic ½MS salt solution supplemented with 250 mM NaCl or 20% (w/v) PEG-6000. The stressed WT and VC plants exhibited retardation of growth, whereas *SbSLSP*–overexpressing plants grew healthier (Figure 3E).

### Ion content analysis and H_2_O_2_ accumulation of *SbSLSP*–overexpressing tobacco plants under stress

**Figure 4:**
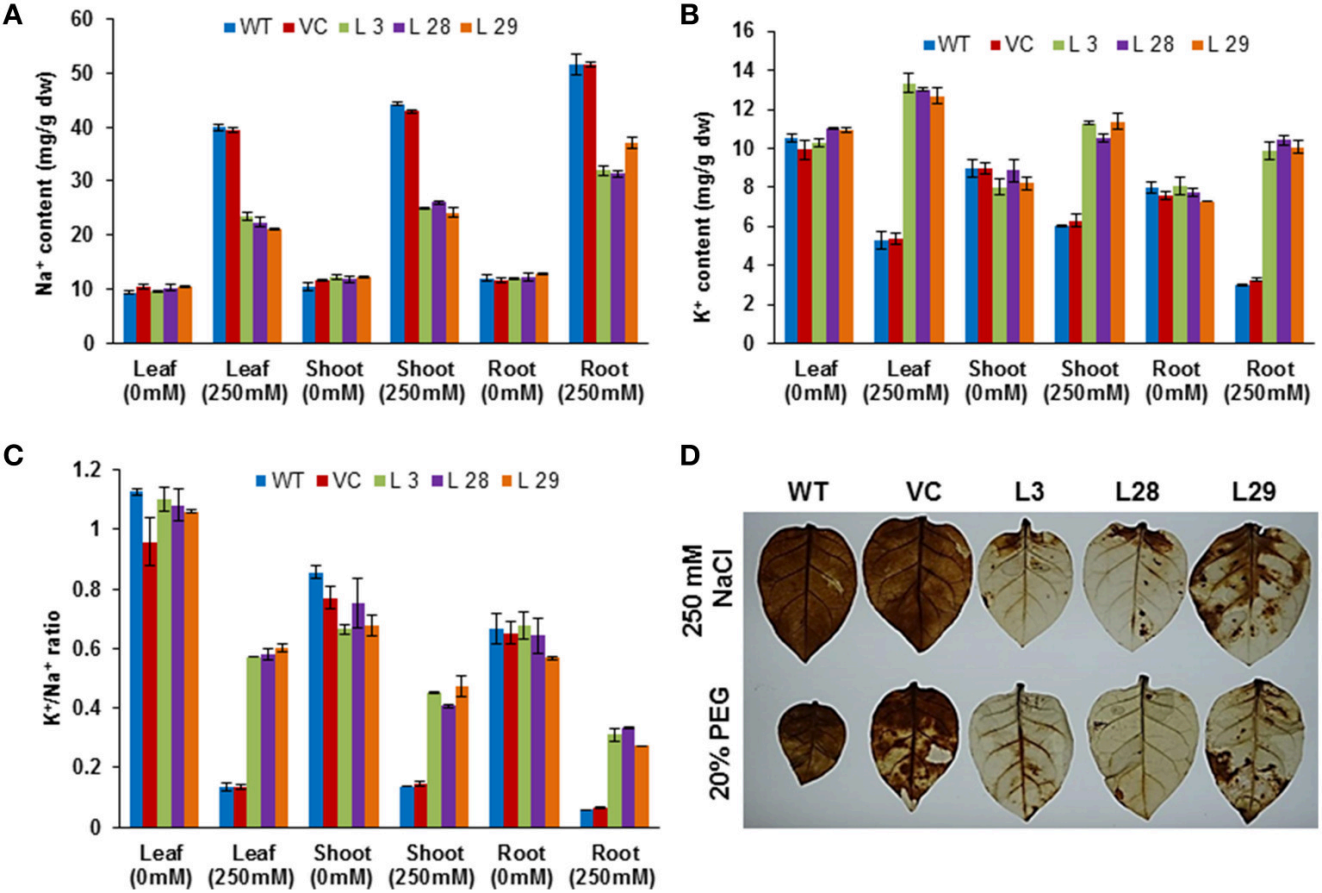
**Ion content and detection of H_**2**_O_**2**_ in T_**2**_ transgenic and wild-type tobacco plants**. **(A)** Na^+^ content in leaves, shoots and roots of transgenic and wild-type plants. **(B)** K^+^ content in leaves, shoots and roots of transgenic and wild-type plants. **(C)** K^+^/Na^+^ ratio with or without salt treatment. Values are means ± SD from three independent trials. **(D)** *In vivo* localization of stress-induced H_2_O_2_ production in leaves of WT, VC and *SbSLSP*–overexpressing transgenic lines after stress treatment by staining with diaminobenzidine.

To determine how SbSLSP maintains the membrane potential in transgenic tobacco plants, we studied the accumulation of K^+^ and Na^+^ ions in tissues of transgenic and WT plants grown under control or salt (250 mM NaCl) treated conditions. The transgenic and WT plants grown under control conditions exhibited almost equal Na^+^ content (Figure 4A). After salinity stress, the Na^+^ ion accumulation was higher in the leaves, stem, and root tissues of the WT and transgenic plants than the plants grown under control conditions, but *SbSLSP–*overexpressing plants accumulated lower Na^+^ compared to WT and VC plants (Figure 4A). An increased K^+^ content was observed in *SbSLSP*–overexpressing plants compared with WT and VC under NaCl stress (Figure 4B). The *SbSLSP*–overexpressing plants showed a significantly improved K^+^/Na^+^ ratio in the leaves, stem, and root tissues relative to WT and VC (Figure 4C). As for accumulation of H_2_O_2_ during stress conditions, histochemical staining of leaves with diaminobenzidine showed more intense staining in WT and VC plants compared to *SbSLSP*–overexpressing plants (Figure 4D).

### Overexpression of the *SbSLSP* gene improves the ROS-scavenging mechanism under salinity and drought stress

**Figure 5:**
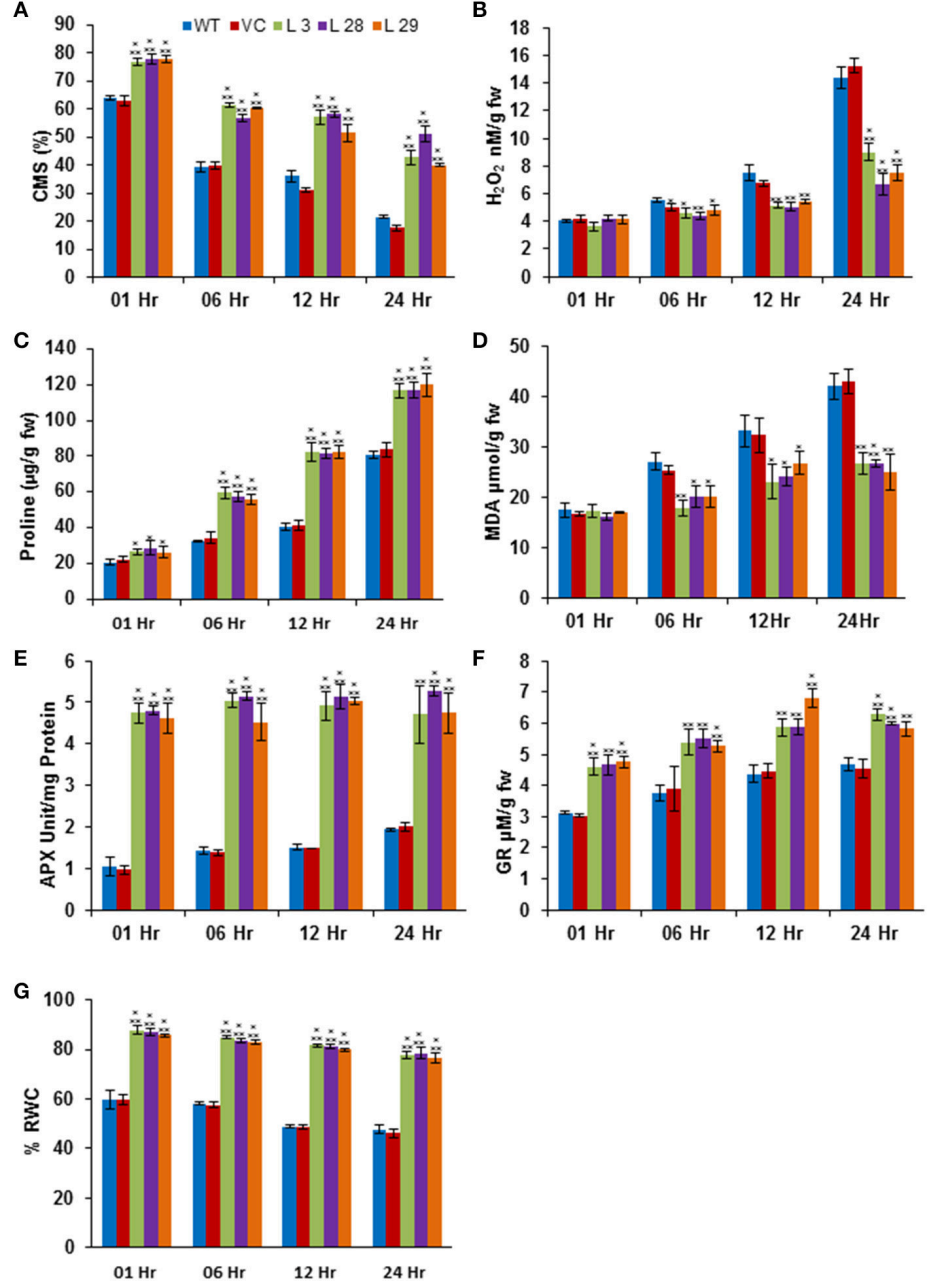
**Biochemical and physiological responses of ***SbSLSP*** transgenic plants, and VC and WT under salinity stress (250 mM NaCl). (A)** Percentage of cell membrane stability. **(B)** Changes in hydrogen peroxide content. **(C)** Changes in the level of proline accumulation. **(D)** Levels of lipid peroxidation expressed in terms of MDA content. **(E)** Changes in ascorbate peroxidase enzyme activity. **(F)** Changes in glutathione reductase enzyme activity. **(G)** Percentage of relative water content. One-way analysis of variance (ANOVA) was used to test the significance between the mean values of control and transgenic plants, and comparison among mean values was performed using ezANOVA (http://www.cabiatl.com/mricro/ezanova/). The difference between control and transgenic lines were statistically significant at ^*^P < 0.05, ^**^P < 0.01 and ^***^P < 0.001.

**Figure 6:**
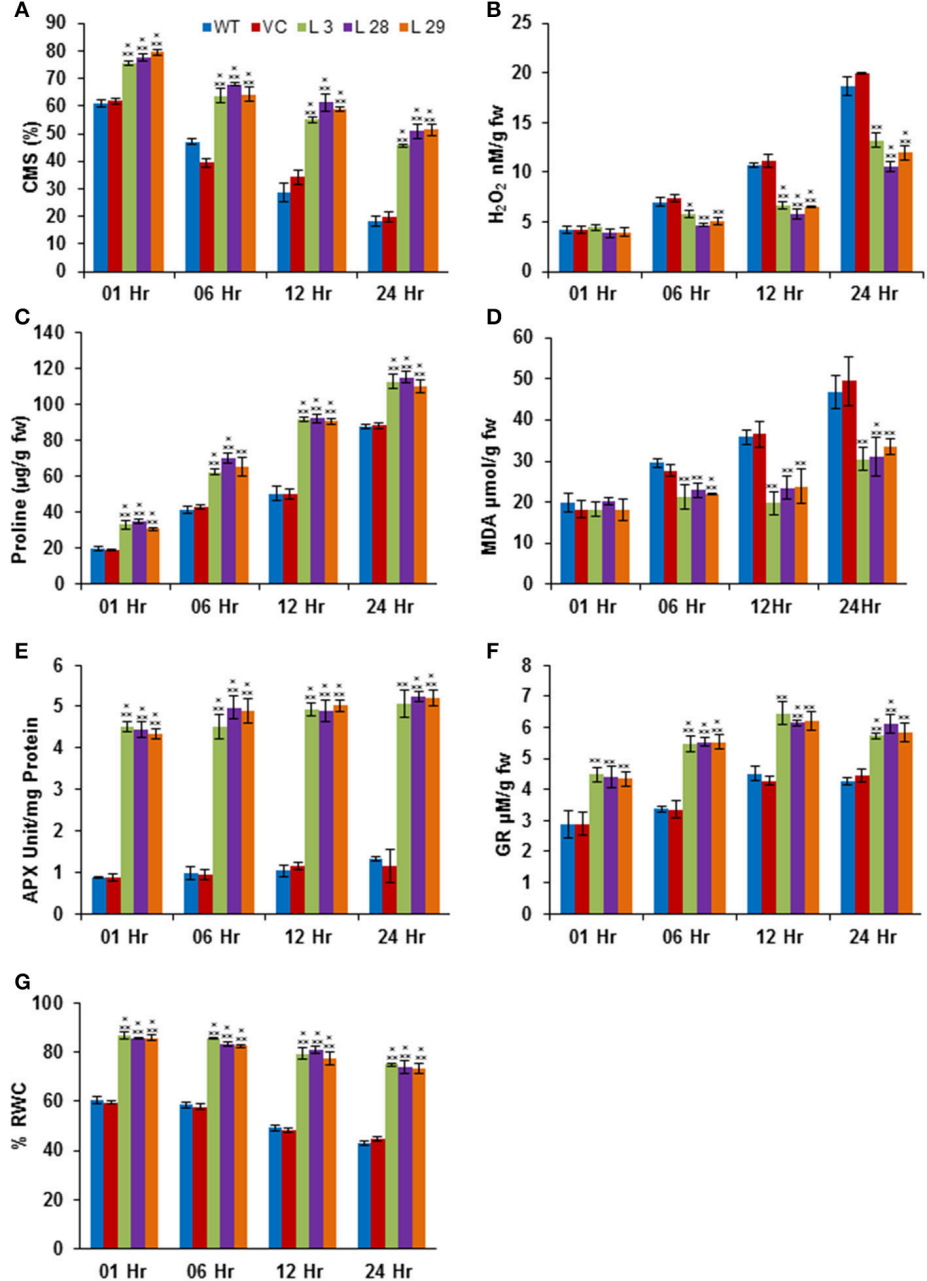
**Biochemical and physiological responses of ***SbSLSP*** transgenic plants, and VC and WT under drought stress (20% PEG). (A)** Percentage of cell membrane stability. **(B)** Changes in hydrogen peroxide content. **(C)** Changes in the level of proline accumulation. **(D)** Levels of lipid peroxidation expressed in terms of MDA content. **(E)** Changes in ascorbate peroxidase enzyme activity. **(F)** Changes in glutathione reductase enzyme activity. **(G)** Percentage of relative water content. One-way analysis of variance (ANOVA) was used to test the significance between the mean values of control and transgenic plants, and comparison among mean values was performed using ezANOVA (http://www.cabiatl.com/mricro/ezanova/). The difference between control and transgenic lines were statistically significant at ^*^P < 0.05, ^**^P < 0.01 and ^***^P < 0.001.

The abiotic stresses exert secondary oxidative stress to plants besides the ionic toxicity, which results in peroxidation of the membrane lipids. The degree of lipid peroxidation, ROS-accumulation and integrity of cell membranes under salinity and drought stresses were determined by quantifying the accumulation of malondialdehyde (MDA) and H_2_O_2_, and the CMS in T_2_ transgenic seedlings. Simultaneously, accumulation of proline was also estimated under same conditions. Under salinity- and drought-stress conditions, there was significantly less accumulation of MDA and H_2_O_2_, and higher CMS and proline in transgenic lines compared to WT and VC plants, confirming reduced damage, higher membrane integrity and better osmotic adjustments of *SbSLSP*–overexpressing plants (Figures 5A–D, 6A–D, respectively). To confirm improved ROS-scavenging mechanisms under stress conditions, the activity of oxidative enzymes was also analyzed. The activity of glutathione reductase (GR, EC 1.6.4.2) and ascorbate peroxidase (APX, EC 1.11.1.11) increased markedly in *SbSLSP*–overexpressing seedlings compared to that of WT and VC plants (Figures 5E,F, 6E,F). Furthermore, a higher relative water content was found in *SbSLSP*–overexpressing seedlings compared to WT and VC seedlings (Figures 5G, 6G). These data indicate improved capacity of osmotic adjustment in transgenic tobacco seedlings.

### Expression of ROS-scavenging and signaling genes in *SbSLSP*–overexpressing tobacco plants

**Figure 7:**
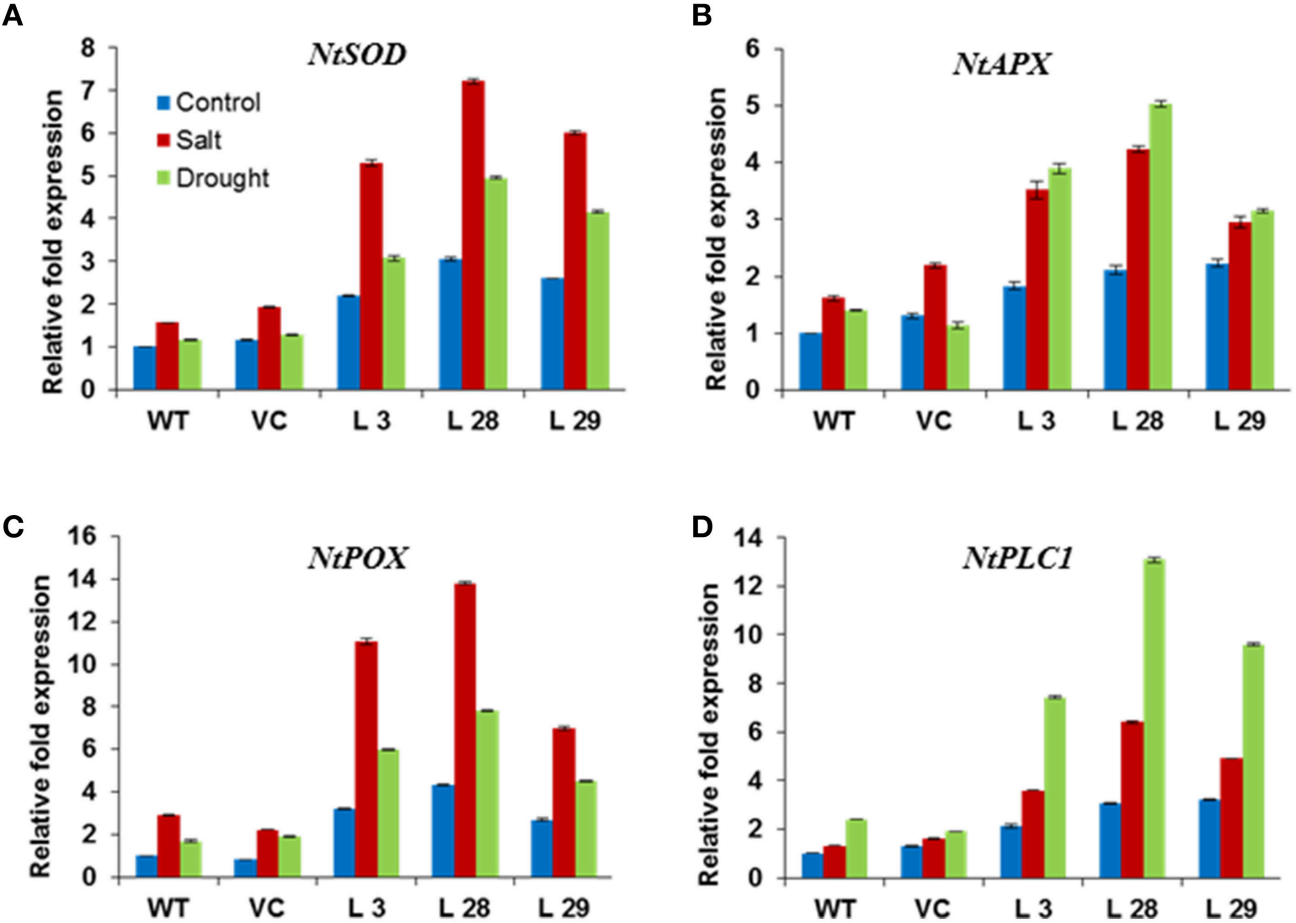
**Expression of ROS-scavenging (A) ***NtSOD***, (B) ***NtAPX***, (C) ***NtPOX***, and signaling (D) ***NtPLC1*** genes in ***SbSLSP*** transgenic plants, and VC and WT plants under salinity and drought stress using real-time PCR**. *Actin* was used as an internal control. Data represents the mean ± SD of three replicates.

To further elucidate the mechanism(s) behind the improved stress tolerance of *SbSLSP*–overexpressing plants, we carried out expression analysis of stress-responsive genes. The transcript levels of ROS-scavenging genes *NtSOD, NtAPX, NtPOX*, and phosphatidic acid mediated signaling pathway enzyme gene *NtPLC1* were higher in *SbSLSP*–overexpressing plants than that of WT and VC plants under both salinity and drought stresses (Figures 7A–D). Under control conditions, the transgenic lines also showed higher expression of ROS-scavenging and *NtPLC1* genes. These results suggest that the *SbSLSP* overexpression regulates the expression of these genes.

## Discussion

High salinity and drought impede crop growth, survival and productivity. Plants adapted to survive stresses via mechanisms that operate at the whole plant, tissue, organ, cellular, and molecular level. Plant's plasticity to salt stress comprises a number of genes encoding proteins which involved in ion homeostasis, osmolyte synthesis, and antioxidant synthesizing enzymes (Agarwal et al., 2013). Being more efficient to survive saline conditions than glycophyte, halophytic plants become a potential bioresource for novel salt-responsive genes. Large numbers of unknown or hypothetical genes are reported in the EST database of many halophytic plants (Yadav et al., 2014). Among the ESTs database of *S. brachiata* (Jha et al., 2009), *SbSLSP* showed higher up-regulation upon salinity stress and desiccation (Figures 1A,B), which was supported by the *in silico* analysis of the putative promoter, containing stress-related *cis*-regulatory elements (Table S2). The expression of *SbSLSP* is controlled via an ABA-mediated signaling pathway, which includes MYB-dependent induction. Besides MYB- and MYC-binding elements, the promoter region showed other stress-responsive elements, including salinity, cold, and heat shock–responsive CAAT boxes (Table S3; Figure S4). It was interesting that a large number of motifs were detected that are responsible for root nodule-specific expression (Table S2). This suggests that, apart from the stress-responsive expression, *SbSLSP* may also play a crucial role in nutrient absorption and nitrogen assimilation.

The NCBI protein blast analysis revealed that the SbSLSP protein has a high sequence identity to a SNARE-like superfamily protein from *Z. marina* and *A. thaliana*. Phylogenetic analysis also revealed that the SbSLSP has close proximity with halophyte *Z. marina* (Figure S2). More closeness of SbSLSP protein with *Z. marina* homolog as compared to Arabidopsis suggests a common plasticity mechanism in halophytes. The SbSLSP also showed high sequence identity (80–95%) with hypothetical/uncharacterized proteins from different plant species. But protein blastp analysis against the Arabidopsis proteins displayed only similarity with the SNARE-like protein. SNAREs are fundamental controllers of membrane trafficking machinery and are playing crucial role in many biological processes such as growth, ion homeostasis, hormone and stress signaling (Lipka et al., 2007; El Kasmi et al., 2013). The SNARE proteins also play vital role to cope-up with the abiotic stresses (Zhu et al., 2002; Mazel et al., 2004; Leshem et al., 2006; Hamaji et al., 2009; Kim and Bassham, 2011; Bao et al., 2012; Uemura et al., 2012; Tarte et al., 2015).

*In silico* analysis predicted an N-myristoylation site at 125–130 amino acid position (GVleNT) in the SbSLSP protein (Figure S1). Myristoylation plays a pivotal role in membrane targeting by anchoring proteins to the membrane, and in abiotic stress signaling in plant responses to environmental stresses. The SbSLSP protein also has a conserved domain for clathrin AP complex small-chain domain. The AP complexes are cytosolic heterotetramers which involve in the assembly of clathrin-coated vesicles (CCVs). The CCVs play crucial role in transportation of membrane proteins via endocytic and secretory pathways (Nakatsu and Ohno, 2003). In the subcellular localization study, the SbSLSP protein mainly localized to the plasma membrane (Figure 1D), which supporting our *in-silico* analysis result. The SbSLSP showed similarity with SNARE-like proteins and has AP complexe small-chain domain. Plants subjected to osmotic–stress, experience alterations in cell membrane dynamics and lipid arrangement to uphold cellular integrity and to reduce ion leakage (Koning et al., 2008). In the present study, the *SbSLSP*–overexpressing transgenic plants experiencing salinity and osmotic stress exhibited higher cell membrane stability hence less leakage. The bulk-flow endocytosis prevents the excessive osmotic water loss by internalization of the additional surface area and also helps to regain the turgor pressure (Staehelin and Newcomb, 2000). The *SbSLSP*–overexpressing transgenic lines showed better water status relative to the WT plants (Figures 5G, 6G). Phosphatidic acid (PA) mediated signaling pathway plays vital role in plant stress responses (McLoughlin et al., 2013). The *SbSLSP* transgenic plants maintained higher transcript levels of phosphoinositide-specific phospholipase C1 (*PLC1*) (Figure 7D), suggesting a role of SbSLSP in the salinity and drought stress tolerance through a phosphatidic acid–mediated signaling pathway. The SbSLSP protein contained a clathrin adaptor complex small-chain domain and an N-myristoylation site, and also enhanced the expression of *PLC1* in transgenic tobacco, and therefore, it is plausible that SbSLSP is involved in the formation of higher number of CCVs with increased level of PtdIns(4,5)P_2_ and function in phosphatidic acid–mediated salinity and drought-stress adaptation by increasing endocytosis trafficking or signaling during ionic and osmotic stress. It is well-known that improved trafficking could more efficiently allocate transporters required for water and ion flux. We observed that the transgenic lines overexpressing *SbSLSP* showed a better water status (Figures 5G, 6G) and lower Na^+^ accumulation with a higher K^+^/Na^+^ ratio (Figures 4A–C), which indicates increased efficiency of transporters.

The *SbSLSP–*overexpressing tobacco plants promoted seed germination and conferred enhanced tolerance to salinity and osmotic stress (Figures 2, 3). Stressed *SbSLSP* transgenic plants had longer primary roots compared to control plants, thus higher absorption area, and therefore they are capable of absorbing more water. This may be the reason for higher RWC and biomass synthesis under water-deficit stress. The improved root morphology of *SbSLSP* transgenic plants permit efficient use of water, and is ultimately contribute to stress tolerance. The higher chlorophyll content, better survival rate and healthier growth of *SbSLSP* transgenic plants during stress assays corroborate the increased stress tolerance of *SbSLSP–*overexpressing tobacco plants. Similar to our study, Tarte et al. (2015) reported that SNARE gene *AtSFT12*-overexpressing Arabidopsis plants showed higher salinity and osmotic tolerance and maintained higher RWC compared to WT plants.

Salt stress–induced optimal Na^+^ and K^+^ levels are essential for cellular health in terms of chlorophyll degradation, membrane lipid peroxidation and leaf senescence (Zhu et al., 2001). The *SbSLSP*-overexpressing tobacco plants accumulated lower Na^+^ and higher K^+^ ions in roots, stems and leaves as compared to WT and VC plants grown in salt stress conditions (Figures 4A–C). *SbSLSP–*overexpressing tobacco plants also showed a significant improvement in the K^+^/Na^+^ ratio (Figure 4C). The above findings suggest that overexpression of *SbSLSP* acts as a safeguard for the cells from stress-induced injuries and premature senescence. It has been reported that SNAREs protein played crucial role in the regulation of ion transport (Leyman et al., 1999).

In addition to Na^+^ toxicity, salinity, and drought also impose membrane disorganization and generation of ROS (Hasegawa et al., 2000). Various physiological and biochemical parameters have been positively correlated with CMS in stress conditions (Verslues et al., 2007; Harb et al., 2010; Foyer and Shigeoka, 2011). In the present study, *SbSLSP* transgenic plants had higher CMS compared to WT and VC plants (Figures 5A, 6A), which is positively correlated with enhanced levels of relative water content and proline. Plants establish their ROS homeostasis in order to minimize oxidative damages to cellular macromolecules (Pitzschke et al., 2009). The *SbSLSP*–overexpressing lines maintained less ROS in term of MDA and H_2_O_2_ levels in response to salt and drought stresses (Figures 5B,D, 6B,D), which indicates reduced oxidative damage. The ROS detoxifying enzymes activity is improved in plants under numerous biotic and abiotic stresses (Baek et al., 2006; Miller et al., 2010). The transcript levels of ROS-scavenging genes *APX, POX* and *SOD* were analyzed in overexpressing lines and WT plants. The *SbSLSP*–overexpressing lines grown under salinity or drought stress conditions maintained higher transcript levels of these genes relative to WT plants (Figures 7A–C). The lower ROS levels in transgenic lines corroborated by the increased transcript levels of ROS-scavenging genes. Bao et al. (2012) also showed that overexpression of SNARE gene *OsSYP71* enhanced oxidative stress tolerance of transgenic rice by upregulation of peroxidase.

**Figure 8:**
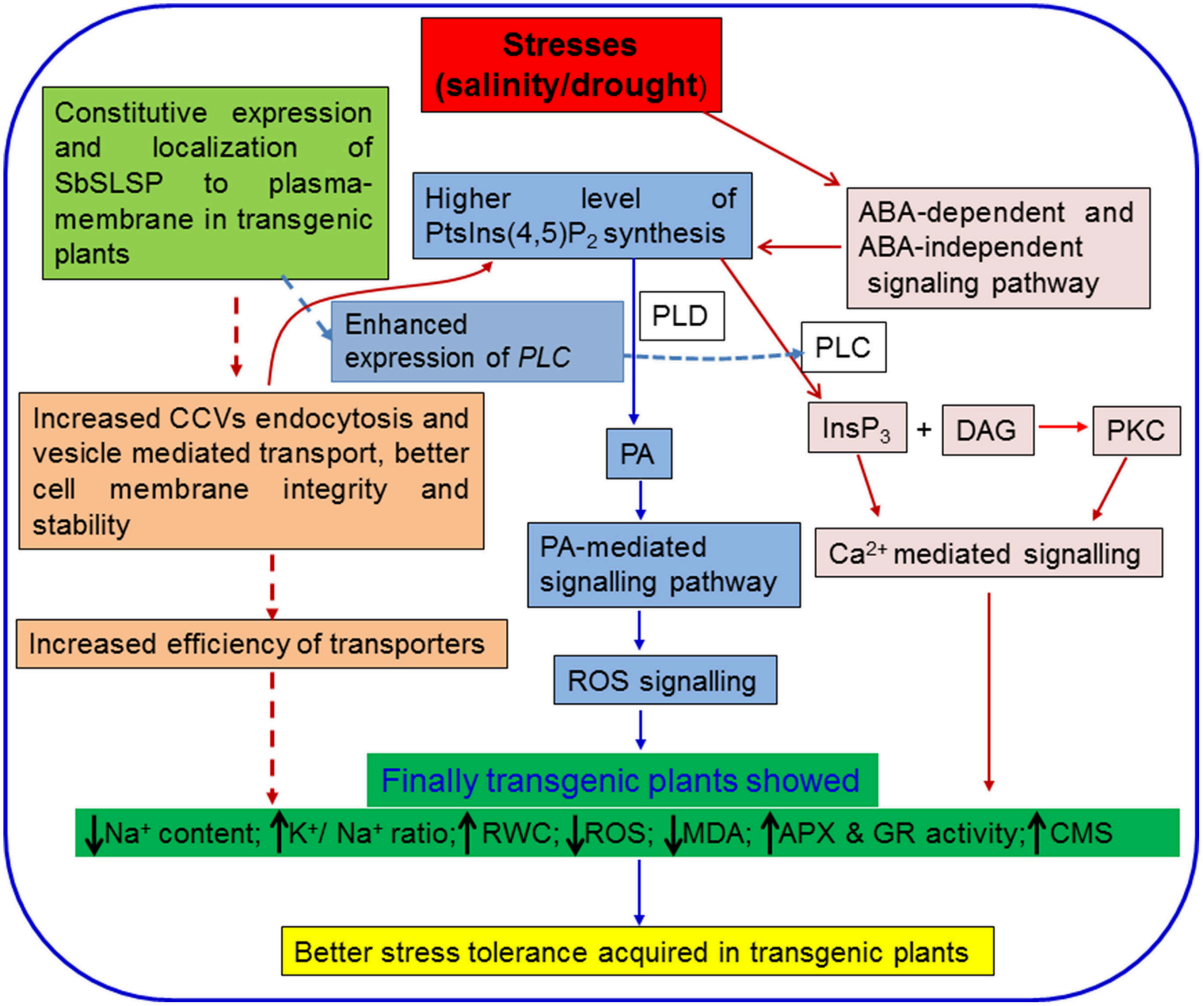
**A hypothetical model for the role of SbSLSP in abiotic stress tolerance**. The model represents the established signaling cascade under abiotic stresses. The hypothesized plausible role of SbSLSP in stress tolerance was represented by broken lines. Constitutive overexpression of SbSLSP protein may leads to increased clathrin coated vesicles mediated endocytosis leading to higher accumulation of PtsIns(4,5)P_2_and then PA mediated stress signaling, which increased the efficiency of transporters and cell membrane stability. The SbSLSP somehow increased the transcript expression of *PLC* gene which may contribute to the increased Ca^2+^ mediated signaling pathway. Conclusively all these pathways converge together and results in better abiotic stress tolerance in the transgenic plants. CCVs: Clathrin coated vesicles; PtsIns(4,5) P_2:_ phosphatidyl-inositol-4,5-bisphosphate; PLC: phospholipase C; PLD: phospholipase D; PA: phosphatidic acid; InsP_3_: inositol-1,4,5-triphosphate; DAG: diacylglycerol; PKC: protein kinase C.

Based on the findings of our present study, we hypothesized that the enhanced salinity and drought tolerance in the *SbSLSP* transgenic plants is associated with the higher RWC, less accumulation of ROS, higher accumulation of osmolytes and low Na^+^ content with a better K^+^/Na^+^ ratio. By combining the earlier reports and findings of the present study, we proposed a hypothetical model, depicting how SbSLSP may have contributed to achieve enhanced stress tolerance in transgenic tobacco (Figure 8). We hypothesized that constitutive overexpression of SbSLSP protein may leads to increased CCVs mediated endocytosis leading to higher accumulation of PtsIns(4,5)P_2_ and then PA mediated stress signaling, which increased the efficiency of transporters and cell membrane stability. The SbSLSP also increased the expression of *PLC* gene, which may contribute to the increased Ca^2+^ mediated stress signaling pathway. Conclusively all these pathways converse and results in better abiotic stress tolerance in the transgenic plants.

In conclusion, the present study is the first investigation into the function of *SbSLSP*, a novel salt-inducible gene from halophyte *S. brachiata*. The promoter region not only has several abiotic stress-responsive motifs, but also many of root nodule–specific motifs, which suggests its role in stress tolerance, as well as in nutrient assimilation. Overexpression of *SbSLSP* resulted in enhanced salinity and drought tolerance, enhanced plant growth, better water status, improved physiological and biochemical parameters, and better regulation of ROS-scavenging genes in response to salt and osmotic stress. Bioengineering of the novel *SbSLSP* gene may allow development of crop plants with improved stress tolerance, and appears to hold great promise in improving agricultural productivity under high salinity and drought stress.

## Author contributions

Conceived and designed the experiments: NY, PA, and BJ. Performed the experiments: DS, NY, and VT. Analyzed the data: DS, NY, and VT. Wrote the paper: NY and BJ. All authors approved the final manuscript.

### Conflict of interest statement

The authors declare that the research was conducted in the absence of any commercial or financial relationships that could be construed as a potential conflict of interest.
