# Supplementary material for: A SNARE-Like Superfamily Protein SbSLSP from the Halophyte Salicornia brachiata Confers Salt and Drought Tolerance by Maintaining Membrane Stability, K+/Na+ Ratio, and Antioxidant Machinery
Source: Front Plant Sci. 2016 Jun 2;7:737. doi: 10.3389/fpls.2016.00737 (PMC4889606; doi:10.3389/fpls.2016.00737)
Supplement: Supplementary file 1 [file Table1.DOCX]

**Table S1. Primers used in this study.**

| **Oligo Name** | **Sequence (5' to 3')** | **Used for** |
| --- | --- | --- |
| GSPR1 | GTTTGCCACATACATCCTTCAA | 5′-RACE |
| GSPR2 | GCAAGTTCGTCATACTCA TCCT |  |
| GSPR3 | AGCGGGAACTCCATTAAACC |  |
| AAP | GGCCACG CGTCGACTAGTAC(G)_16_ |  |
| AUAP | GGCCA CGCGTCGACTAGTAC |  |
| SbSLSPF | CCCAAGCTTATGTTGCTGGCAGTATTGAT | Full length cloning |
| SbSLSPR | CCGCTCGAGTCAAAACTCAGTTGGGGG |  |
| SbSLSPPR1 | CTCAGCGGGAACT CCATTAAACC | Promoter region cloning |
| SbSLSPPR2 | GGAAATCAATACTGCCAGCAACATC |  |
| SbSLSPPR3 | TAGAGAGAGAAAGAGAAGCTAAAGA |  |
| SbSLSP_RTF1 | GGCAAGGATGAGTATGACGAA | qRT-PCR |
| SbSLSP_RTR1 | AACTCCCGTCCAAACAATTTC |  |
| BTF | GGAGTCACCGAGGCAGAG | qRT-PCR (*β*-*tubulin* as internal control) |
| BTR | ATCACATATCAGAAACCACAA |  |
| SbSLSPCAF | CACCATGTTGCTGGCAGTATTGAT | Sub-cellular localization study |
| SbSLSPCAR | TCAAAACTCAG TTGGGGG |  |
| SbSLSPPF | AACAGGGCCCATGTTGCTGGCAGTATTGAT | *SbSLSP* cloning in pCAMBIA2301 |
| SbSLSPPR | ATCGGGGTACCTCAAAACTCAGTTGGGGG |  |
| SbSLSP_RTF2 | GCTGTCCTTGGAGATGTTCA | Transgenic screening and RT-PCR |
| SbSLSP_RTR2 | CCAAACAATTTCATCAAG GCAT |  |
| QACTF | CGTTTGGATCTTGCTGGTCGT | qRT-PCR (*actin* as internal control) |
| QACTR | CAGCAATGCCAGGGAACATAG |  |
| NtPLCF | CTGATGACGGA GCAGGTGT | qRT-PCR |
| NtPLCR | CTGAGCAAGTTCCGCTGAG |  |

Note: The underlined sequences denotes the restriction enzymes recognition sites used for cloning
