# Supplementary material for: A SNARE-Like Superfamily Protein SbSLSP from the Halophyte Salicornia brachiata Confers Salt and Drought Tolerance by Maintaining Membrane Stability, K+/Na+ Ratio, and Antioxidant Machinery
Source: Front Plant Sci. 2016 Jun 2;7:737. doi: 10.3389/fpls.2016.00737 (PMC4889606; doi:10.3389/fpls.2016.00737)
Supplement: Supplementary file 2 [file Table2.DOC]

**Table S2:** Different *cis-*regulatory motifs identified on *SbSLSP* putative promoter by PLACE and PlantCARE online programs

| **Category** | **Name of *cis*-element** | **Function** | **Sequence** | **Position (upstream of ATG)** |
| --- | --- | --- | --- | --- |
| Abiotic stress inducible | ANAERO1CONSENSUS | One of the 16 anaerobically induced motifs | AAACAAA | -22 (+), -141 (+), -261 (-) |
|  | CCAATBOX1 | CCAAT box found in the promoter of heat shock transcription factor gene | CCAAT | -167 (-), -321 (+), -604 (+) |
|  | CURECORECR | Copper and oxygen responsive element | GTAC | -240 (+), -240 (-), -790 (+), -790 (-) |
|  | EBOXBNNAPA | E-box, R response element | CANNTG | -108 (+), -108 (-), -236 (+), -236 (-), -548 (+), -548 (-), |
|  | GT1GMSCAM4 | Pathogen and salt inducible GT-1 element found in soybean (Glycine max) | GAAAAA | -669 (+) |
|  | LTRE1HVBLT49 | Low temperature responsive element (LTRE-1) in barley | CCGAAA | -844 (+) |
|  | LTRECOREATCOR15 | Core sequence of cold, drought and ABA responsive element found in promoter of cor15a gene | CCGAC | -748 (+) |
|  | MYB1AT | MYB protein binding site in promoter of dehydration responsive element | WAACCA | -816 (+) |
|  | MYB1LEPR | Binding site of regulator of defence-related genes | GTTAGTT | -95 (+) |
|  | MYB2AT | Dehydration responsive MYB2 binding site | TAACTG | -483 (-) |
|  | MYB2CONSENSUSAT | Dehydration responsive MYB2 binding site | YAACKG | -209 (+), -483 (-), -548 (+) |
|  | MYBCORE | MYB1 and MYB2 binding site, dehydration responsive and MYB.Ph3 mediated regulation of flavonoid biosynthesis | CNGTTR | -116 (-), -209 (-), -483 (+), -548 (-) |
|  | MYBST1 | Core motifs of potato myb binding site | GGATA | -569 (+) |
|  | MYCCONSENSUSAT | Consensus sequence of dehydration responsive MYC2 and ICE1 binding site | CANNTG | -108 (+), -108 (-), -236 (+), -236 (-), -548 (+), -548 (-), |
|  | PREATPRODH | Proline and hypoosmolarity responsive elements in promoter of Proline dehydrogenase gene | ACTCAT | -105 (-) |
| Conserved motifs | BOXIINTPATPB | Conserved Box II motifs of NCII promoters of plastids | ATAGAA | -426 (-), -470 (-), -567 (+), -635 (-) |
|  | CAATBOX1 | CAAT consensus sequence of pea legA gene | CAAT | -167 (-), -320 (+), -438 (+), -538 (+), -603 (+), -770 (-) |
|  | CTRMCAMV35S | CT rich element of 35S promoter downstream to transcription start site | TCTCTCTCT | -34 (+) |
|  | HEXAMERATH4 | Hexamer motif found in promoter of histone 4 gene | CCGTCG | -872 (-) |
|  | LEAFYATAG | WUSCHEL-tyope homoebox protein binding site | CCAATGT | -169 (-) |
|  | POLASIG1 | Putative polyadenylation signals in nuclear genes of higher plants | AATAAA | -154 (-), -535 (-), -449 (-) |
|  | POLASIG2 | Polyadenylation site found in rice alpha-amylase gene | AATTAAA | -266 (-) |
|  | POLASIG3 | Consensus sequence for plant polyadenylation site | AATAAT | -185 (-), -211 (-), -286 (+), -293 (-), -302 (+) |
|  | TATABOX3 | TATA box of sweet potato sporamin A gene | TATTAAT | -436 (-) |
|  | TATABOX4 | TATA box of sweet potato sporamin A and beta-phaseolin gene promoter | TATATAA | -614 (+) |
|  | TATABOX5 | TATA box of glutamine synthetase gene | TTATTT | -510 (+), -448 (+), -292 (+), -153 (+), -303 (-) |
| Light responsive | CGACGOSAMY3 | Coupling element for G box element | CGACG | -872 (+) |
|  | GATABOX | GATA box found in promoter region of LHCII proteins and regulate their light responsive expression | GATA | -180 (-), -272 (-), -568 (+), -591 (+), -737 (+) |
|  | GT1CONSENSUS | Light regulated binding site of GT-1 protein | GRWAAW | -73 (-), -150 (-), -151 (-), -182 (-), -669 (+) |
|  | GT1CORE | Core sequence of GT-1 binding element | GGTTAA | -817 (-) |
|  | HDZIP2ATATHB2 | Binding site of light responsive negative auto regulatory Homeobox gene ATHB-2 | TAATMATTA | -187 (-) |
|  | IBOXCORE | I-box, light regulated conserved upstream sequence found in bath upstream and downstream | GATAA | -181 (-) |
|  | INRNTPSADB | Inr element found in light responsive genes and regulate transcription initiation by TATA box independent manners | YTCANTYY | -255 (+), -371 (+), -401 (+), -406 (+), -411 (+), -416 (+), -460 (+), -465 (+), -490 (+), -495 (+) |
|  | LBOXLERBCS | L- Box, found in promoter of light regulated rbcS gene | AAATTAACCAA | -820 (+) |
| Miscellaneous | DOFCOREZM | Maize Dof protein or protein with Dof domain binding site, involve in various stress and developmental stages | AAAG | -10 (+), -17 (+), -37 (-), -48 (-), -59 (+), -199 (+), -229 (-), -269 (-), -277 (-), -343 (+), -450 (-), -520 (+), -666 (+), -835 (+) |
|  | WBBOXPCWRKY1 | Elicitor-induced and zinc dependent expression | TTTGACY | 197 (+), 211 (+) |
|  | WBOXATNPR1 | W-box recognised by salicyalic acid-induced WRKY DNA binding proteins | TTGAC | 198 (+), 212 (+) |
|  | WBOXHVISO1 | Sugar-responsive WRKY transcription factor binding site | TGACT | 88 (+), 199 (+), 213 (+) |
|  | WBOXNTERF3 | W-box found in the promoter region of a transcriptional repressor ERF3 gene in tobacco | TGACY | 88 (+), 199 (+), 213 (+) |
| Phytohormone inducible | ARR1AT | Non-symbiotic haemoglobin 2 gene promoter expressed by cytokinin mediate ARR1 binding | NGATT | -329 (-), -503 (+), -513 (+), -558 (+), -726 (+), -772 (+), -810 (-) |
|  | GAREAT | Gibberellin responsive element | TAACAAR | -98 (-), -192 (-) |
|  | MYBGAHV | Central element of gibberellin responsive complex, gibberellin regulated expression of myb in aleurone layer | TAACAAA | -192 (-) |
|  | NTBBF1ARROLB | Tobacco Dof protein binding site in Agrobacterium required for tissue specific and auxin induced expression | ACTTTA | -200 (-) |
|  | P-BOX | Gibberellin responsive element | GCCTTTTGAGT | -65 (-) |
| Tissue specific expression | -300ELEMENT | Endosperm specific expression of glutein gene | TGHAAARK | -670 (+) |
|  | AACACOREOSGLUB1 | Core sequenceof AACA motifs for endosperm specific expression of rice glutein gene | AACAAAC | -193 (-) |
|  | CACTFTPPCA1 | Mesophyll cells specific expression in C4 plants | YACT | -218 (-), -159 (+), -429 (+), -598 (+), -589 (+), -616 (-), -640 (-), -760 (-) |
|  | GCN4OSGLUB1 | "GCN4 motif" for endosperm specific expression | TGAGTCA | -785 (-) |
|  | NODCON1GM | Consensus sequence of nodulin specific expression | AAAGAT | -271 (-), -452 (-) |
|  | NODCON2GM | Consensus sequence of nodulin specific expression | CTCTT | -230 (+), -596 (+), -839 (+) |
|  | OSE1ROOTNODULE | Root specific expression in the nodules of infected cells | AAAGAT | -271 (-), -452 (-) |
|  | OSE2ROOTNODULE | Root specific expression in the nodules of infected cells | CTCTT | -39 (+), -230 (+), -596 (+) |
|  | POLLEN1LELAT52 | Pollen activation of expression in tomato | AGAAA | -24 (+), -36 (-), -471 (-), -636 (-), -711 (+) |
