# Supplementary material for: A SNARE-Like Superfamily Protein SbSLSP from the Halophyte Salicornia brachiata Confers Salt and Drought Tolerance by Maintaining Membrane Stability, K+/Na+ Ratio, and Antioxidant Machinery
Source: Front Plant Sci. 2016 Jun 2;7:737. doi: 10.3389/fpls.2016.00737 (PMC4889606; doi:10.3389/fpls.2016.00737)
Supplement: Supplementary file 3 [file Table3.DOC]

**Table S3:** Number of abiotic stress responsive *cis*-regulatory motifs identified on putative promoter sequence of *SbSLSP* gene using PLACE and PlantCARE online programs

| **Motif type** | **Name** | **Sequence** | **Number of motif** |
| --- | --- | --- | --- |
| Anaerobic stress | ANAERO1CONSENSUS | AAACAAA | 2 (+), 1 (-) |
| Drought (osmotic) stress | EBOXBNNAPA | CANNTG | 3 (+), 3 (-) |
|  | LTRECOREATCOR15 | CCGAC | 1 (+) |
|  | MYB1AT | WAACCA | 1 (+) |
|  | MYB2AT | TAACTG | 1 (-) |
|  | MYB2CONSENSUSAT | YAACKG | 2 (+), 1 (-) |
|  | MYBCORE | CNGTTR | 1 (+), 3 (-) |
|  | MYCCONSENSUSAT | CANNTG | 3 (+), 3 (-) |
|  | PREATPRODH | ACTCAT | 1 (-) |
| Metal stress | CURECORECR | GTAC | 2 (+), 2 (-) |
| Salinity stress | GT1GMSCAM4 | GAAAAA | 1 (+) |
| Temperature stress | CCAATBOX1 | CCAAT | 2 (+), 1 (-) |
|  | LTRE1HVBLT49 | CCGAAA | 1 (+) |
|  | LTRECOREATCOR15 | CCGAC | 1 (+) |

Note: The “+” sign denotes the cloned promoter sequence while “-“ sign to the complementary sequence
