# Supplementary material for: A SNARE-Like Superfamily Protein SbSLSP from the Halophyte Salicornia brachiata Confers Salt and Drought Tolerance by Maintaining Membrane Stability, K+/Na+ Ratio, and Antioxidant Machinery
Source: Front Plant Sci. 2016 Jun 2;7:737. doi: 10.3389/fpls.2016.00737 (PMC4889606; doi:10.3389/fpls.2016.00737)
Supplement: Supplementary file 4 [file Presentation1.PPT]

## Slide 1
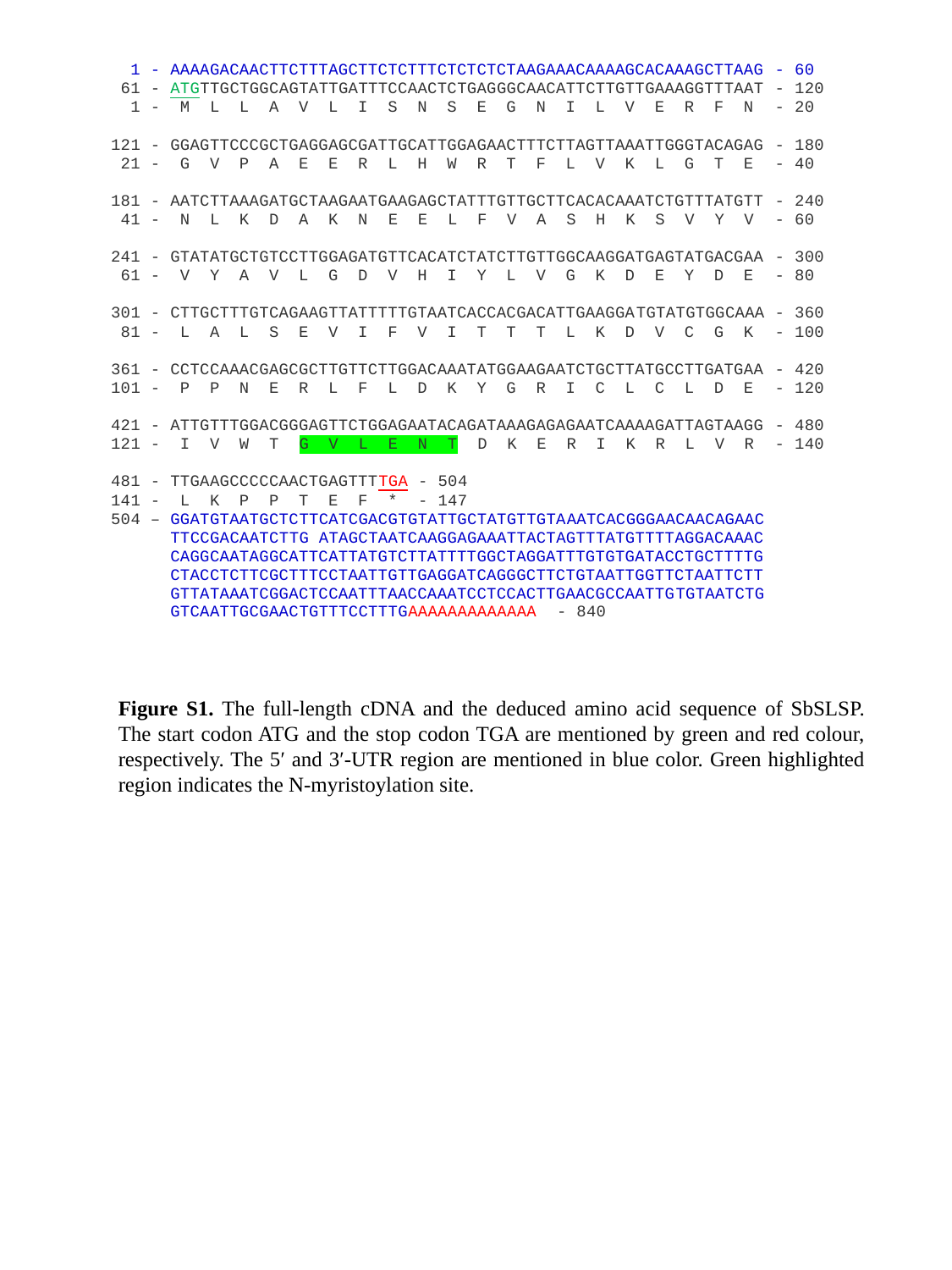

Figure S1. The full-length cDNA and the deduced amino acid sequence of SbSLSP. The start codon ATG and the stop codon TGA are mentioned by green and red colour, respectively. The 5′ and 3′-UTR region are mentioned in blue color. Green highlighted region indicates the N-myristoylation site.
