# Supplementary material for: A SNARE-Like Superfamily Protein SbSLSP from the Halophyte Salicornia brachiata Confers Salt and Drought Tolerance by Maintaining Membrane Stability, K+/Na+ Ratio, and Antioxidant Machinery
Source: Front Plant Sci. 2016 Jun 2;7:737. doi: 10.3389/fpls.2016.00737 (PMC4889606; doi:10.3389/fpls.2016.00737)
Supplement: Supplementary file 5 [file Presentation2.PPTX]

## Slide 1
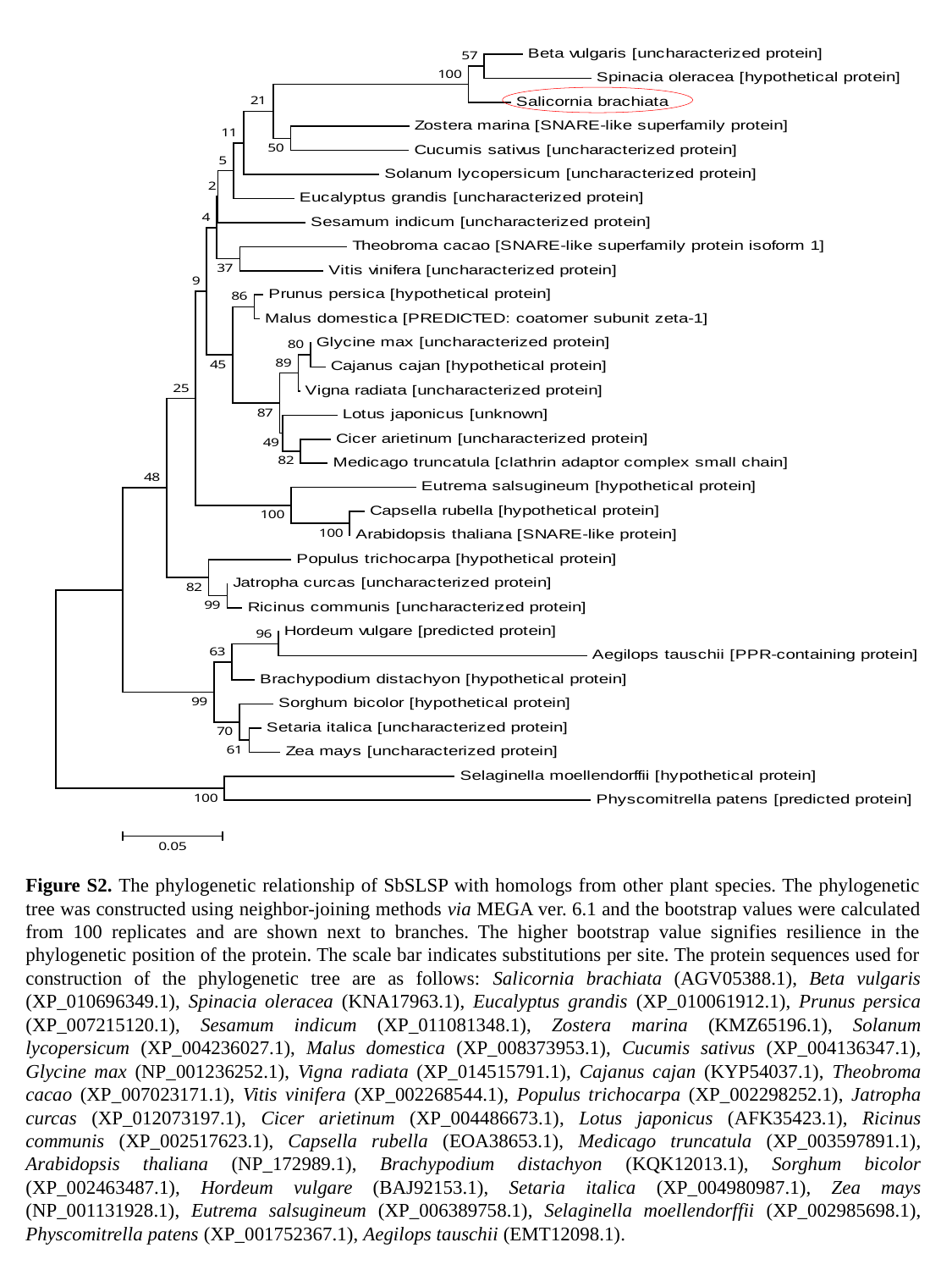

Figure S2. The phylogenetic relationship of SbSLSP with homologs from other plant species. The phylogenetic tree was constructed using neighbor-joining methods via MEGA ver. 6.1 and the bootstrap values were calculated from 100 replicates and are shown next to branches. The higher bootstrap value signifies resilience in the phylogenetic position of the protein. The scale bar indicates substitutions per site. The protein sequences used for construction of the phylogenetic tree are as follows: Salicornia brachiata (AGV05388.1), Beta vulgaris (XP_010696349.1), Spinacia oleracea (KNA17963.1), Eucalyptus grandis (XP_010061912.1), Prunus persica (XP_007215120.1), Sesamum indicum (XP_011081348.1), Zostera marina (KMZ65196.1), Solanum lycopersicum (XP_004236027.1), Malus domestica (XP_008373953.1), Cucumis sativus (XP_004136347.1), Glycine max (NP_001236252.1), Vigna radiata (XP_014515791.1), Cajanus cajan (KYP54037.1), Theobroma cacao (XP_007023171.1), Vitis vinifera (XP_002268544.1), Populus trichocarpa (XP_002298252.1), Jatropha curcas (XP_012073197.1), Cicer arietinum (XP_004486673.1), Lotus japonicus (AFK35423.1), Ricinus communis (XP_002517623.1), Capsella rubella (EOA38653.1), Medicago truncatula (XP_003597891.1), Arabidopsis thaliana (NP_172989.1), Brachypodium distachyon (KQK12013.1), Sorghum bicolor (XP_002463487.1), Hordeum vulgare (BAJ92153.1), Setaria italica (XP_004980987.1), Zea mays (NP_001131928.1), Eutrema salsugineum (XP_006389758.1), Selaginella moellendorffii (XP_002985698.1), Physcomitrella patens (XP_001752367.1), Aegilops tauschii (EMT12098.1).
