# Supplementary material for: A SNARE-Like Superfamily Protein SbSLSP from the Halophyte Salicornia brachiata Confers Salt and Drought Tolerance by Maintaining Membrane Stability, K+/Na+ Ratio, and Antioxidant Machinery
Source: Front Plant Sci. 2016 Jun 2;7:737. doi: 10.3389/fpls.2016.00737 (PMC4889606; doi:10.3389/fpls.2016.00737)
Supplement: Supplementary file 6 [file Presentation3.ppt]

## Slide 1
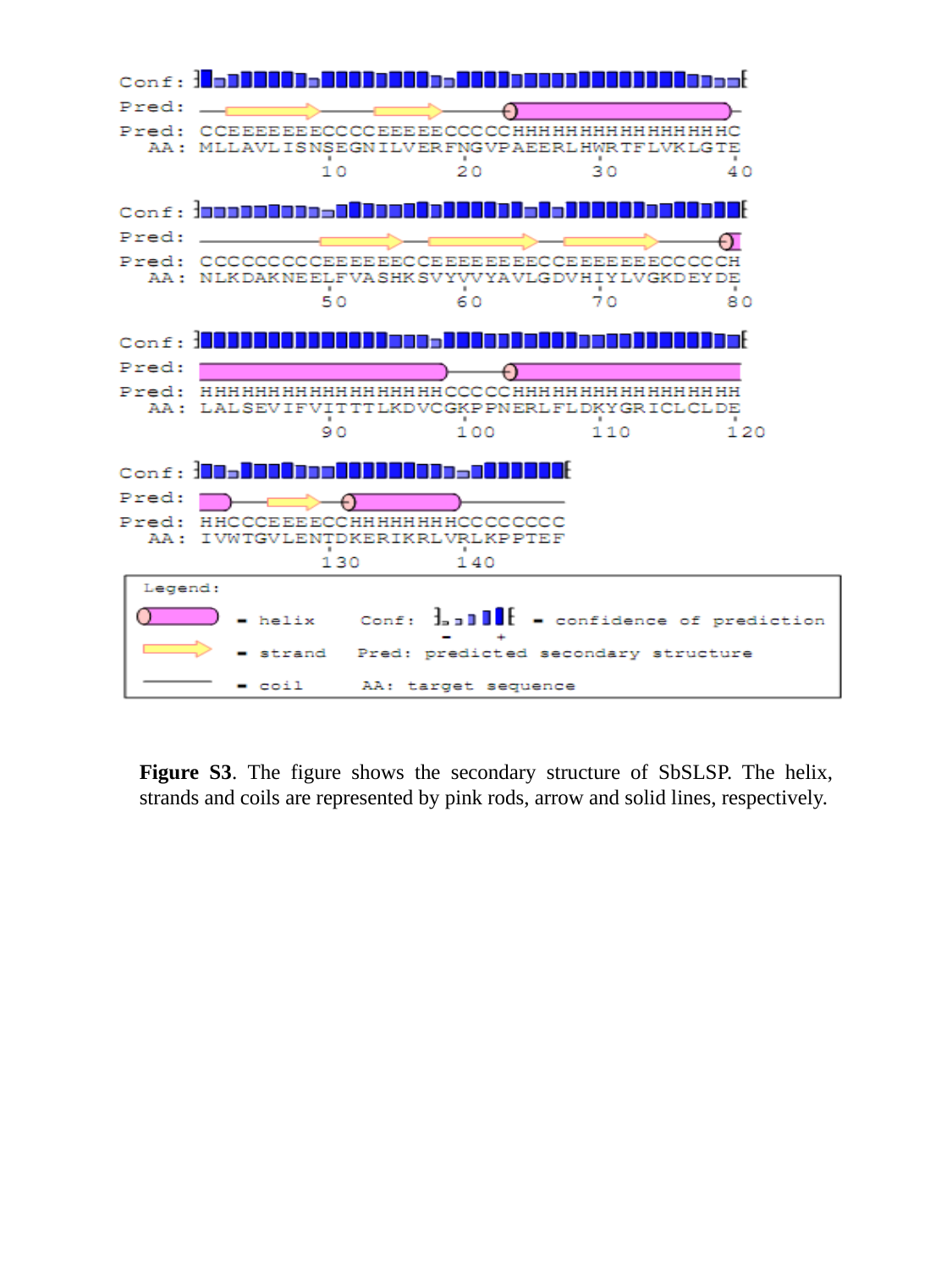

Figure S3. The figure shows the secondary structure of SbSLSP. The helix, strands and coils are represented by pink rods, arrow and solid lines, respectively.
