# Supplementary material for: A SNARE-Like Superfamily Protein SbSLSP from the Halophyte Salicornia brachiata Confers Salt and Drought Tolerance by Maintaining Membrane Stability, K+/Na+ Ratio, and Antioxidant Machinery
Source: Front Plant Sci. 2016 Jun 2;7:737. doi: 10.3389/fpls.2016.00737 (PMC4889606; doi:10.3389/fpls.2016.00737)
Supplement: Supplementary file 7 [file Presentation4.PPT]

## Slide 1
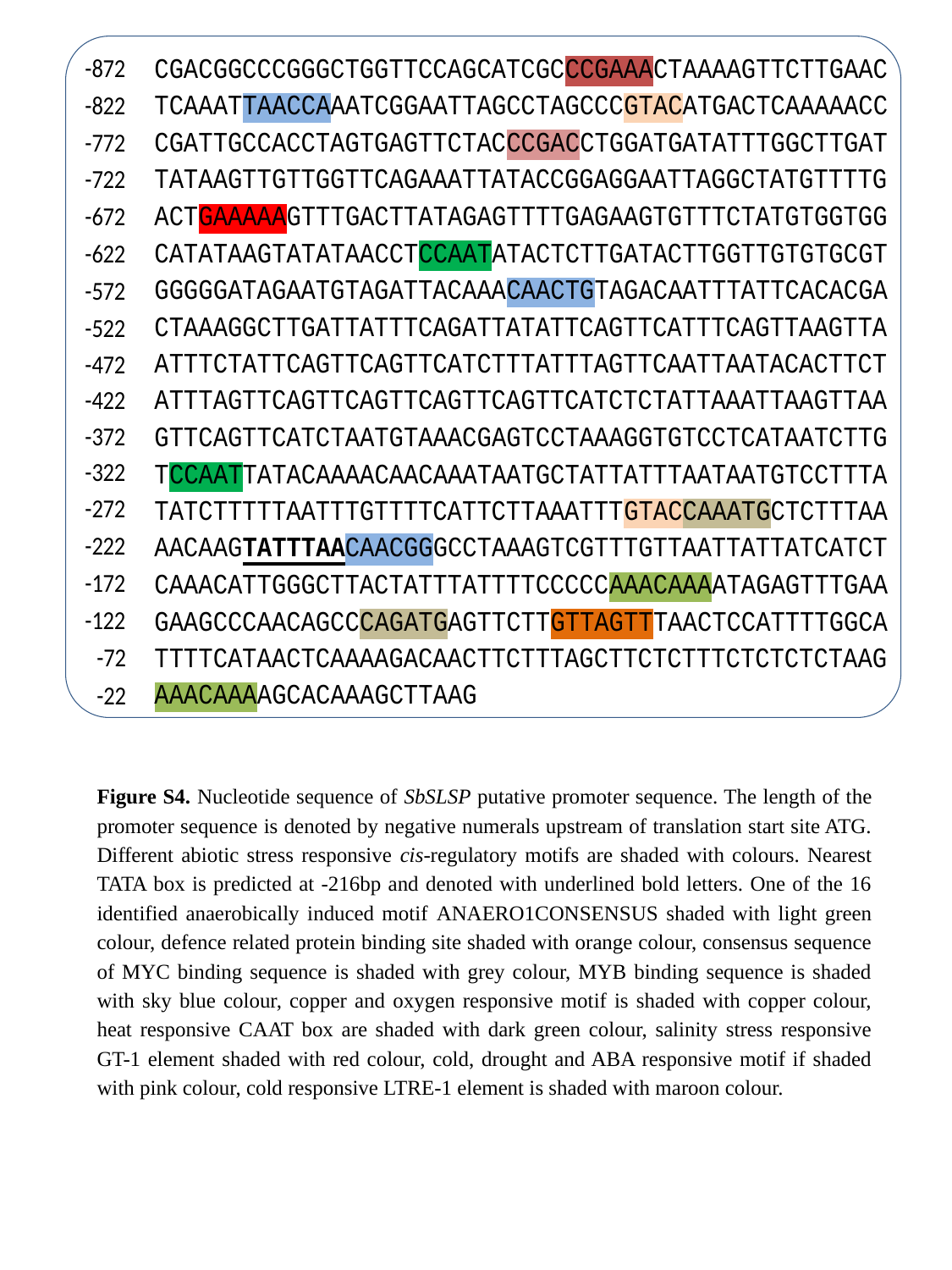

Figure S4. Nucleotide sequence of SbSLSP putative promoter sequence. The length of the promoter sequence is denoted by negative numerals upstream of translation start site ATG. Different abiotic stress responsive cis-regulatory motifs are shaded with colours. Nearest TATA box is predicted at -216bp and denoted with underlined bold letters. One of the 16 identified anaerobically induced motif ANAERO1CONSENSUS shaded with light green colour, defence related protein binding site shaded with orange colour, consensus sequence of MYC binding sequence is shaded with grey colour, MYB binding sequence is shaded with sky blue colour, copper and oxygen responsive motif is shaded with copper colour, heat responsive CAAT box are shaded with dark green colour, salinity stress responsive GT-1 element shaded with red colour, cold, drought and ABA responsive motif if shaded with pink colour, cold responsive LTRE-1 element is shaded with maroon colour.
